# Precision cardiovascular medicine: shifting the innovation paradigm

**DOI:** 10.3389/fsci.2025.1474469

**Published:** 2025-10-07

**Authors:** Masanori Aikawa, Abhijeet R. Sonawane, Sarvesh Chelvanambi, Takaharu Asano, Arda Halu, Joan T. Matamalas, Sasha A. Singh, Shizuka Uchida, Elena Aikawa, Alex Arenas, Jean-Luc Balligand, Chiara Giannarelli, Calum A. MacRae, Neil V. Morgan, Cécile Oury, Hendrik Tevaearai Stahel, Joseph Loscalzo

**Affiliations:** 1 Center for Interdisciplinary Cardiovascular Sciences, Division of Cardiovascular Medicine, Department of Medicine, Brigham and Women’s Hospital, Harvard Medical School, Boston, MA, United States,; 2 Center for Excellence in Vascular Biology, Division of Cardiovascular Medicine, Department of Medicine, Brigham and Women’s Hospital, Harvard Medical School, Boston, MA, United States,; 3 Channing Division of Network Medicine, Department of Medicine, Brigham and Women’s Hospital, Harvard Medical School, Boston, MA, United States,; 4 Center for RNA Medicine, Department of Clinical Medicine, Aalborg University, Copenhagen, Denmark,; 5 Department of Computer Engineering and Mathematics (DEIM), Rovira I Virgili University (URV), Tarragona, Spain,; 6 Institute of Experimental and Clinical Research, Pole of Pharmacology and Therapeutic, Cliniques Universitaires St-Luc, Université Catholique de Louvain, Brussels, Belgium,; 7 NYU Cardiovascular Research Center, Division of Cardiology, Department of Medicine, Department of Pathology, New York University Grossman School of Medicine, New York, NY, United States,; 8 Division of Cardiovascular Medicine, Department of Medicine, Brigham and Women’s Hospital, Harvard Medical School, Boston, MA, United States,; 9 Department of Cardiovascular Sciences, School of Medical Sciences, College of Medicine and Health, University of Birmingham, Birmingham, United Kingdom,; 10 Laboratory of Cardiology, GIGA-Cardiovascular Sciences, University of Liège, Liège, Belgium,; 11 Bern University Hospital, University of Bern, Bern, Switzerland,; 12 Department of Medicine, Brigham and Women’s Hospital, Harvard Medical School, Boston, MA, United States

**Keywords:** cardiovascular disease, cardiology, precision medicine, systems biology, network medicine, heterogeneity, artificial intelligence

## Abstract

Despite the development of potent drugs for modifiable risk factors and advances in mechanistic biomedical research, cardiovascular diseases (CVDs) collectively remain the leading cause of death globally, indicating a need for new, more effective therapies. A foundational challenge is the multilevel heterogeneity that characterizes CVDs—from their complex pathobiological mechanisms at the molecular and cellular levels, to their clinical presentations and therapeutic responses at the individual and population levels. This variability arises from individuals’ unique genomic and exposomic characteristics, underscoring the need for precision approaches. Other key challenges include the long navigation times, high costs, and low success rates for drug development, often compounded by the poor “druggability” of new targets. In this article, we explore how these challenges have inspired novel technologies that offer promise in improving health outcomes globally through an integrative precision medicine approach. Key to this transformation is the use of systems biology and network medicine, whereby the application of artificial intelligence to “big data”, ranging from clinical information to unbiased multiomics (e.g., genomics, transcriptomics, proteomics, and metabolomics) can elucidate disease mechanisms, yield novel biomarkers for disease progression, and identify potential drug targets. In parallel, new computational approaches are helping translate these discoveries into novel therapies and overcome druggability barriers. The transition to a precision-based research and innovation paradigm in cardiovascular medicine will require greater interdisciplinary collaboration, data science implementation at every stage, and new partnerships between academia and industry. Global policy leadership is also essential to implement suitable models of research funding and organization, data infrastructures and policies, medicines regulations, and patient access policies promoting equity.

## Introduction: challenges are opportunities

“The greater the obstacle, the more glory in overcoming it.”

Molière

Challenges are opportunities. Clinical problems and unanswered questions in cardiovascular medicine have driven enormous global efforts aiming to understand the underlying causes of cardiovascular disease (CVD) (1–10). Such needs have also triggered a series of highly valuable technological innovations. Investigation into cardiovascular research in its current form began over a century ago (11–14). In the last several decades, our community has focused on educating the public about lifestyle modifications and dietary interventions to prevent or manage CVD (15). The successful development of effective medicines, such as statins and the newer proprotein convertase subtilisin/kexin type 9 (PCSK9) inhibitors, has significantly contributed to reducing the incidence of certain cardiovascular conditions (16–19). Additionally, advances in basic science have identified various molecules and pathways, including interleukin (IL)-1β and IL-6, which highlight the role of inflammation beyond traditional modifiable cardiovascular risk factors (20).

Despite these efforts and remarkable advancements, a substantial risk persists for many patients (17, 21–23). While some therapies, such as glucagon-like peptide-1 receptor (GLP-1R) agonists, can reduce the burden of chronic diseases (24, 25), there is still a dire need to identify additional strategies to address the complex interplay across these diseases. While there is a benefit to optimal treatment of type 2 diabetes in terms of being able to reduce the excess mortality risk associated with CVD, this effect was only seen in patients with no previous diagnoses of CVD. This suggests a strong rationale for fine-tuning therapeutic strategies to combat complex diseases (26). From 2010 to 2019, the number of deaths caused by hypertension increased among adults aged 35–64 in 86.2% of counties in the United States (27). The burden of CVD is projected to grow over the next few decades. The number of elderly patients with calcific aortic valve stenosis, a major complication of chronic kidney disease, is projected to more than double by 2050 in the United States and Europe (28). Overall, CVD continues to be the leading cause of death globally (17), resulting in 19 million deaths worldwide in 2020. This toll increased by 18.7% in the past decade and will likely rise to 26 million by 2030 (28–30).

Difficult problems require innovative solutions. One of the foundational challenges that complicates CVD treatment is the heterogeneity of the complex pathobiological mechanisms and clinical presentations of CVDs. High-throughput, multimodality, multiomics data have ushered in a new era of big data in CVD research, with each type of data introducing a new layer of complexity to the existing challenge of identifying novel mechanisms and biomarkers (31). Addressing these numerous challenges is necessary for advancing precision CVD medicine.

In this article, we explore how key challenges in cardiovascular medicine have inspired novel technologies that offer promise in improving health outcomes globally. We illustrate how systems biology, network medicine, and artificial intelligence (AI), supported by technological innovations in omics, are providing meaningful biological insights into the complexity of disease heterogeneity—fostering new discoveries and targets for precision medicine interventions. We then discuss computational drug discovery and new classes of medicines that may overcome the limitation of conventional small-molecule drugs. Finally, we outline the interdisciplinary and intersectoral actions, underpinned by global policy leadership, necessary to implement a precision-based paradigm for innovation in cardiovascular medicine globally.

## Current challenges in CVD research

### Biology of heterogeneity in CVD

**FIGURE 1:**
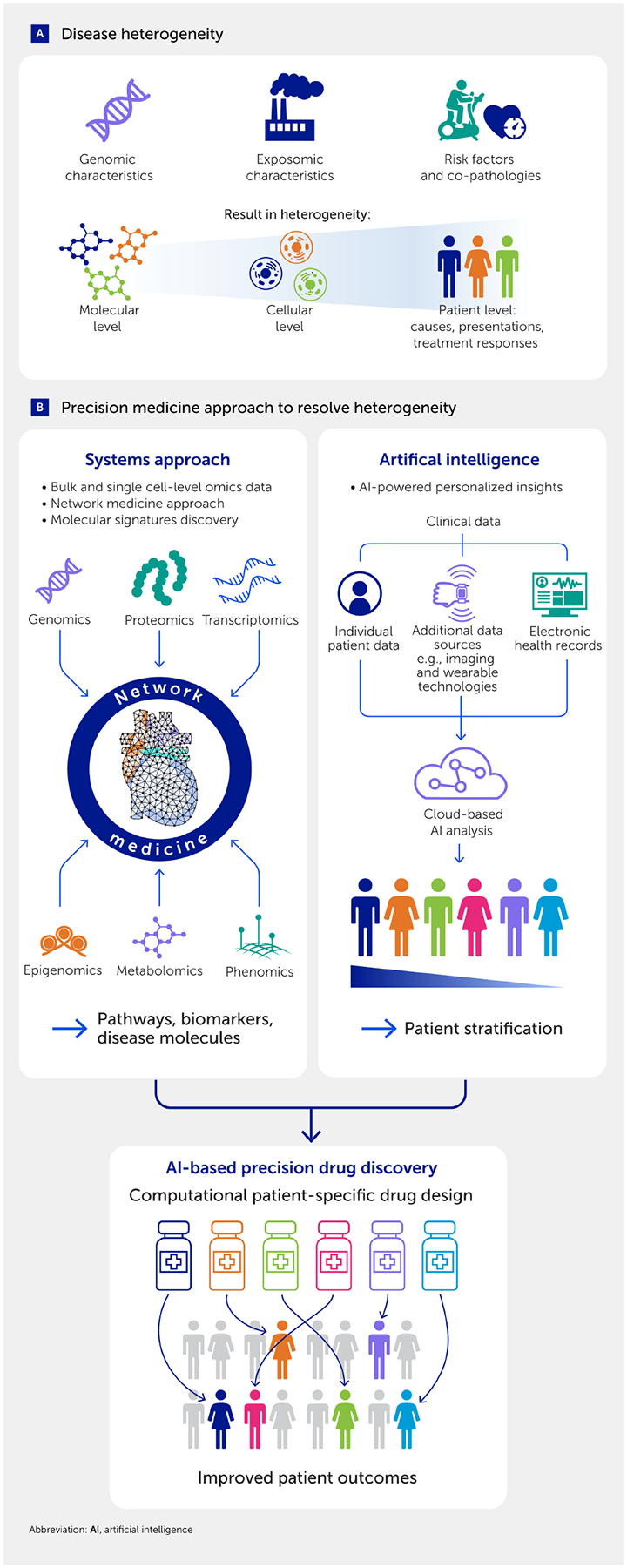
Precision cardiovascular medicine as a solution to heterogeneity in cardiovascular diseases (CVDs). **(A)** The heterogeneity in CVDs arises due to many different factors and manifests at multiple levels. **(B)** A multipronged systems approach leads to precision medicine. On one front, the integration of multiomics data using network medicine techniques unveils molecular pathways and disease biomarkers. Simultaneously, an artificial intelligence (AI)-powered approach utilizes clinical data to offer translational endpoints, such as patient stratification and the development of precision drugs and therapies. Together, these approaches hold the potential to significantly enhance patient outcomes.

CVD heterogeneity is evident in the diversity observed in clinical presentations, underlying causes, and responses to treatments. This variability arises from individuals’ unique characteristics at the genomic (e.g., single-nucleotide polymorphisms) and exposomic (e.g., air pollution or pesticides) levels (32–37) (Figure 1A), underscoring the need to discover new mechanisms and drug targets to advance precision cardiovascular medicine (38). Multiomics approaches have provided more detailed explanations of how such genomic or exposomic signatures contribute to the development of complex diseases (32–36, 39). Recent evidence suggests that human pathobiology is also heterogeneous at multiple levels, from cellular responses to disease mechanisms and clinical features (Figure 1A). Such heterogeneity presents major challenges that have hindered our ability to gain a comprehensive understanding of disease mechanisms and develop more effective therapies for complex human diseases, which have proven to be even more intricately mechanistic than traditionally thought (35, 38, 40–44). Between 1954 and 1977, epidemiological findings of the renowned Framingham Heart Study established risk factors for coronary heart disease and stroke, with a particular emphasis on dyslipidemia and hypertension (45–47). Notably, elevated low-density lipoprotein cholesterol (LDL-C) became a viable target for lowering the incidence of CVD events primarily via statin therapy. Despite the efficacy of statins in reducing LDL-C levels and the incidence of major CVD events, a significant residual risk remains (48).

Clinical, epidemiological, and genetic evidence also points to the presence of phenotypic heterogeneity within CVDs, encompassing conditions such as myocardial infarction, angina pectoris, hypertrophic cardiomyopathy, and heart failure (49–54). For example, genomic mutations in cytochrome P450 2C (CYP2C9), solute carrier anion transporter family 1B1 (SLCO1B1), and adenosine triphosphate (ATP)-binding cassette super-family G member 2 (ABCG2) impact the response to drugs such as statins (55, 56). Additionally, interacting pathologies can lead to CVD, including patients with a predisposition to venous or arterial thrombosis—there is even evidence of familial clustering in some cases, leading to inherited hypercoagulable states. Genetic, environmental, and other phenotypic modifiers can also contribute to this group of conditions, making the diagnosis and management of these disorders particularly challenging (57). Many pathways that contribute to human diseases influence disease severity by interacting within a large and complex biological network of genes, proteins, and signaling pathways.

In parallel with clinical evidence, findings from single-cell RNA sequencing datasets have suggested that each cell type associated with CVD, such as macrophages, represents a heterogeneous population (58–66). The balance between subsets of immune cells (e.g., pro- vs. anti-inflammatory macrophage subpopulations) in a local microenvironment, such as the atherosclerotic plaque, may determine the risk of disease progression or the onset of clinical complications (e.g., myocardial infarction) (67, 68). As various new technologies capable of unbiased screening for target discovery continue to emerge (e.g., plasma proteomics of clinical samples and single-cell RNA sequencing of cells within cardiovascular tissues or blood), subsequently generated datasets become larger and more complex (69, 70). To identify promising new targets from large datasets analyzing complex factors, we must involve analytic platforms capable of processing the vast amounts of data generated (71).

### Complex pathobiological mechanisms for CVD

CVDs are complex entities shaped by multiple factors. It has become increasingly clear that simply focusing on a single molecule or pathway does not allow a comprehensive understanding of the complex interactions and interdependencies among molecules and pathways acting within a larger biological system. Despite their phenotypic diversity, CVDs exhibit common underlying pathophysiologies. This is seen in conditions such as atherosclerosis, which is shared among vascular diseases such as coronary and peripheral artery diseases (CAD and PAD, respectively). The progression of atherosclerosis, however, involves different components of the immune system. CVDs—including heart failure, arrhythmias, hypertension, cardiomyopathies, and thrombosis/embolism—may exhibit varying degrees of complex pathobiological mechanisms that involve factors such as immune response, lipid metabolism, neurohormonal activation, structural variations, sympathetic nervous system modulation, and endothelial dysfunction (72).

Along with lifestyle factors and environmental factors, genetic heterogeneity also plays an important role in complex disease phenotype. Modern technologies such as whole-genome, whole-exome, and targeted sequencing provide sequence information of DNA bases, giving insights into genetic variation. Both allelic and locus heterogeneity can contribute to the development of CVD (43). Large biobanks and databases of multi-ancestry genetic studies now play a crucial role in advancing our understanding of CVDs and thus aid the precision medicine approach (73, 74). While the traditional linear biology approach has been valuable for dissecting individual mechanisms and driving critical discoveries, it does not allow a comprehensive understanding of complex disease pathobiology (75). Preclinical research is powered by many studies built on cell lines and inbred mouse strains (76, 77). The availability of these tools has enabled many mechanistic advances but has failed to capture the heterogeneity observed in the patient population. The use of large animal models and outbred mice could be an important consideration for a systems biology approach. Furthermore, the use of human primary cells to capture the heterogeneity of cells (68) could bring new insights into factors governing inflammation and disease progression. Multi-organ-on-a-chip technologies could be used to mimic the complex cross-organ interactions that regulate cardiovascular diseases (78, 79). These approaches could be bolstered by the utilization of patient-derived induced pluripotent stem cells (iPSCs) reprogrammed into diseased cell types (e.g., endothelial cells, cardiomyocytes, and macrophages) which in turn could be used to perform clinical-trials-on-a-chip for screening patient specific drugs (80, 81). Developing new treatments informed by a holistic view of a complex biological system requires a transition toward a more integrated approach, involving unbiased omics data at multiple levels (e.g., epigenetic, transcriptomic, and proteomic) from the same samples. The identification and understanding of the underlying processes that govern the clinical outcomes are fundamental to the success of precision medicine.

### Long navigation time, high costs, and low success rates for drug development

Other major challenges in cardiovascular medicine include the timelines and costs for target discovery and drug development. Estimated development times for new drugs, from target discovery to launch, range from 5 to 20 years—averaging at 9.1 years for innovative medicines (e.g., first-in-class drugs) (82, 83). However, development times for RNA-targeted therapeutics are generally shorter (82, 84, 85). During the COVID-19 pandemic, open science approaches that mobilized the sharing of data and ideas between academic institutions, the pharmaceutical industry, and governmental institutions, helped accelerate the development and implementation of RNA therapeutics (86, 87).

Low success rates of new drugs in clinical development present another major hurdle. Approximately 90% of drugs fail between their entry into phase I trials and regulatory approval (88, 89). If preclinical drug candidates are included, success rates fall below 10%. The most common cause of failure is the lack of anticipated effects, with the exception of genetic disorders, followed by toxicity/side effects, poor pharmacokinetic parameters, and poor overall clinical development strategies (88–91). For example, hidden drug cardiotoxicity can lead to the discontinuation of clinical trials as well as the withdrawal of drugs post-approval (92). The conventional drug discovery approach that targets a single causal factor also shows limited effectiveness in finding new drugs for complex human diseases, as they involve multiple and overlapping molecular pathways and pathologies. This is, in part, due to this approach relying on a simplified hypothesis for a drug target. Such simplistic hypotheses are often tested using monoclonal cell lines and inbred mice, models that do not fully recapitulate the heterogeneity of complex chronic diseases in humans. Phenotype-driven drug discovery, as opposed to target-based strategies, can address diseases for which mechanisms remain incompletely understood and has been widely used in the pharmaceutical industry (93). Network analysis-powered prediction of the potential impact of each candidate target in human disease may also limit the failure of new drugs at the clinical development stage to a certain extent, owing, in part, to its ability to predict off-target adverse effects (94–96). The benefits of such a comprehensive systems approach to drug discovery ultimately lower the overall costs of development and yield a more efficacious and safe therapeutic agent. These approaches can predict the toxicity profiles of drugs, which in turn avoids the selection of compounds that may fail in later drug development stages due to harmful effects (97, 98).

Following the development of numerous drugs targeting previously identified causal proteins, the need to assess the potential ability of a novel target to be modulated positively or negatively by treatments, colloquially known as “druggability,” remains a key challenge in the development of new drugs (99, 100). Fortunately, technological advances have provided solutions to this challenge. The accumulation of biological and chemical data and the rapid evolution of high-performance computing have enabled the development of various computational strategies, including AI, and helped to design new, effective compounds or predict the potential effects of existing drugs via phenotypic screening. The use of such new technologies and novel computational approaches in drug discovery has opened possibilities for making traditionally undruggable targets druggable (101–103). New platforms, particularly those focused on RNA-targeted therapeutics, also offer precise methods for modulating previously undruggable targets while also reducing development timelines and costs (84, 104–106).

### Precision cardiovascular medicine: through systems biology

The essence of the solution to these challenges is the concept of precision medicine (Figure 1). The National Institutes of Health and the Food and Drug Administration of the United States define precision medicine as an innovative approach that considers individual differences among patients. The goal is to use the right treatments in the right patients at the right time. Each individual possesses unique genomic characteristics, experiences distinct exposures (i.e., environmental factors), and exhibits various combinations of traditional risk factors (e.g., dyslipidemia, hypertension, diabetes, lifestyle, diet, and sex). Moreover, the same stimuli and therapies may elicit heterogeneous responses in different individuals. Considering the specific characteristics of each patient to tailor medical care and interventions with the goal of optimizing treatment effectiveness and minimizing adverse effects is key to precision medicine (107). This necessitates an approach that enables comprehensive molecular profiling, fosters integration to comprehend complex interactions among diverse components, and facilitates the development of predictive models for diseases and biological processes. Systems biology serves as a valuable framework for fulfilling these objectives: it is an interdisciplinary field where the central tenet is that the behavior of a biological system as a whole arises from the complex interplay between its constituent parts (leading to emergent system properties), which cannot be fully understood by studying them individually (108, 109). Below we discuss different aspects of systems biology and potential solutions to challenges in CVD research.

## A systems approach to unraveling the biology of heterogeneity

As discussed, CVD arises from diverse factors affecting complex molecular networks. Such complex mechanisms imply that a traditional reductionist approach to exploring a single cause of disease in the average population, testing a linear hypothesis focused on a single target, and developing a “one-size-fit-all” medicine is overly simplistic (75) and may account for reduced efficacy in a substantial group of patients for which the approved drug has been developed (110). This major challenge has driven our efforts to develop a more holistic, integrative systems approach involving unbiased omics analyses, bioinformatics, and network science to establish precision medicine (Figure 1B) (38, 40, 111, 112). The generation of large amounts of biomedical data, or “big data”, ranging from clinical information in electronic health records (EHRs) to the molecular measurement of analytes using different omics platforms (e.g., genomics, transcriptomics, proteomics, and metabolomics) has also prompted the need for analytical frameworks that can holistically interrogate disease pathobiology. Studying CVDs using multiple omics modalities also requires a systems approach because of the involvement of not only complex tissues, including the heart, vasculature, and valves, but also various common underlying mechanisms—termed endophenotypes—including inflammation, immunity, thrombosis, fibrosis, and calcification.

A systems approach enables the construction of integrated models through the concurrent analysis of entities from different omics layers (e.g., genes, proteins, and metabolites) involved in each disease (Figure 1B). Combining different interacting units (e.g., genes and metabolites) into networks enables the identification of key molecular components and the nature of their interrelationships (e.g., regulatory or co-expression) (112). Similarly, AI and machine learning can pinpoint the key combinations of genomic features that are predictive of disease progression (Figure 1B). A systems approach thus enables simultaneous identification of biomarkers for disease progression and potential drug targets. This includes assessing off-target effects and mechanisms of action, enhancing the translational value of these investigations. Over the past two decades, systems-based research has been effectively applied to cellular systems to reveal a wide variety of emergent biological functions. This approach has complemented and addressed the limitations of the reductionist paradigm prevalent in biomedicine, especially following the influx of high-throughput data during the post-genomic era (109, 113–116). The incorporation of clinical data and disease etiology using AI and an advanced systems approach also allows for better patient stratification into groups based on drug responsiveness, a central tenet of the precision medicine approach (Figure 1B).

### Network medicine for multiomics data integration

Networks form the cornerstone of the systems approach in biomedicine (109, 117) and have been used to analyze rich omics data generated in the past few decades. Being multifactorial, complex chronic diseases, CVDs have benefited greatly from the application of systems and network medicine (38, 118, 119). Currently, a myriad of network-based approaches are readily applicable to a vast array of high-throughput molecular, interaction, and ontological data that are publicly available. These approaches are instrumental in inferring key molecules, subnetworks, and pathways related to CVD, providing invaluable information that may help to better identify novel drug targets for preclinical and clinical testing (120). Network methods have been built and implemented for gene regulation (121–125), protein–protein interactions (126–131), and metabolic interplay (132) to shed new light on the drivers of CVDs. Genetic risk loci identified by genome-wide association studies (GWAS) on features such as the PR interval (133), QRS duration (134), and atrial fibrillation (135, 136) have been studied using gene networks. The structural properties of omics-derived networks help in identifying CVD phenotypes that correlate with network features such as functional modules in CVD (137, 138), congenital heart disease (139), cardiac development, hypertrophy, and heart failure (140). Network medicine has helped identify candidate disease genes for CAD (121, 141) or CVD in general (142). Analyzing proteomic data with the assistance of network-based approaches has revealed the global impact of macrophage activation in vascular disease (94) and helped identify the mechanisms behind indoxyl sulfate-triggered pro-inflammatory macrophage activation (95). Similarly, pathway network analysis allows us to study macrophage activation through PCSK9 (143) and in vein graft disease (144), vascular calcification (145), and rheumatic heart valve disease (146). Simultaneous analysis of global transcriptomics and proteomics of calcific aortic valve disease has revealed important associations with various inflammatory diseases (129). Moreover, horizontal integration of the same omics datatypes measured under different conditions or sources reveals correlated features in various layers. For example, integration of proteomics from valvular interstitial cells—either in two-dimensional (2D) cultures on flat surface or three-dimensional (3D) models on hydrogels—with their extracellular vesicles showed correlated proteins relevant to calcification (147). The unbiased proteomics and systems biology of abdominal aortic aneurysms of mouse models and patients revealed potential novel mechanisms (148). Integrative omics analyses have also been used to study blood pressure regulation and hypertension (149, 150). Parallel to the advances on the omics front, the use of systems pharmacology in CVD has gained prominence (151–155).

### The systems approach to drug discovery

**FIGURE 2:**
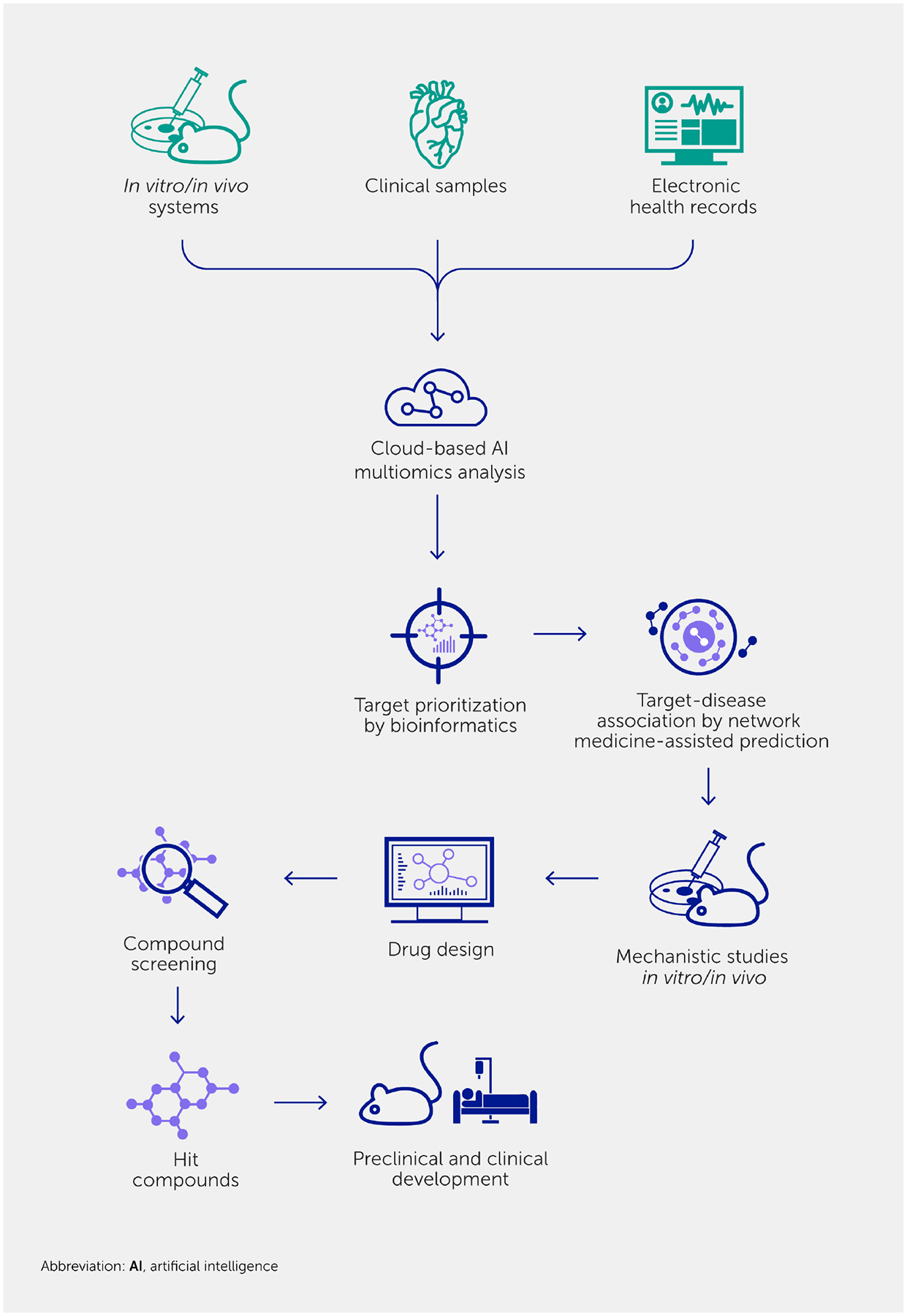
A multilayer systems approach to target discovery and drug development. The hypothesis-driven, reductionist approach may have contributed to low success rates of new therapies based on conventional, basic science-driven clinical development. A systems approach involving unbiased omics, followed by bioinformatics for target prioritization and network medicine-assisted prediction of clinical impact, may facilitate the process and increase the success rate of identifying new targets. Comprehensive *in vitro* and *in vivo* experiments substantiate new concepts. Such models enable earlier implementation of drug design than conventional models.

**FIGURE 3:**
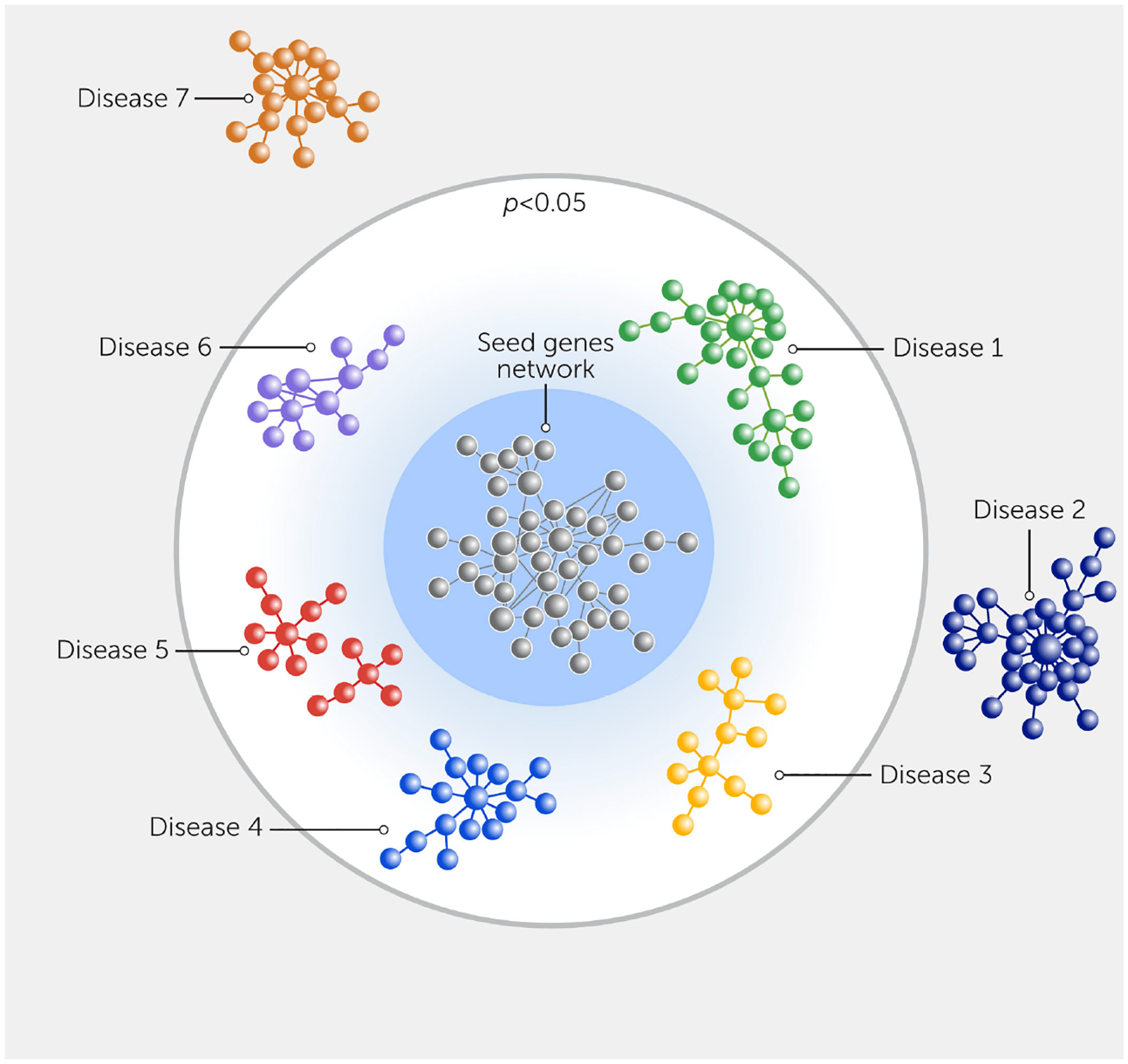
Network prediction of the clinical impact of seed genes on various diseases. The network proximity of the seed genes from analyses (gray), which could be obtained from differentially expressed genes or proteins and various disease gene modules obtained using databases. The *p* value indicates the significance of proximity to the given disease module and other disorders, as compared with random expectation.

Systems biology—involving unbiased omics screening, bioinformatics, and network analysis—has facilitated the discovery of both diagnostic and prognostic biomarkers for CVD. A key strength of this approach is its ability to discover promising targets with improved accuracy in predicting drug efficacy in patients (Figure 2) (38, 94, 95, 144, 156–158). This approach has also enabled us to identify potential targets with a higher likelihood of clinical significance through the integration of different omics datasets and holistic analysis of the disease. Using a systems biology approach, we can find connections between drug target genes and a potentially beneficial clinical outcome (94, 112, 159, 160) (Figure 3). Analyzing which proteins have showcased a shared tendency to change within the human interactome predicted potential regulators of macrophage activation (161). Protein network databases also help to identify proteins closely associated with a node or disease target by “proximity” that can be targeted by new or repurposed drugs to interfere with the disease network. Investigating pathways, key driver genes (162), and network modules associated with a potential drug target can provide key information to researchers by allowing them to choose appropriate cell culture methods, animal models, or even patient stratification in clinical trials.

### Future perspectives in the systems approach to cardiovascular medicine

One of the important promises of systems medicine is its focus on the patient rather than the disease. Specific genes and individual disease-causing mutations can contribute to an individual’s apparent monogenic CVD phenotype, such as cardiomyopathies. However, in some cases, “additive” modifier genes may also play a role, paving the way for precision genetic medicine (163). For example, a previous study aimed to identify genetic drivers of dilated cardiomyopathy, a diagnosis of exclusion among cardiomyopathies (164). After examining 51 curated genes, they identified 19 that showed high evidence but could only explain a minority of cases, suggesting the need for further studies to unearth the mechanism of disease development. We predict, however, the realization of precision medicine goals at scale in the next decade. The implementation of network-based approaches has accelerated research on integrative single-cell omics (165) and spatially resolved omics (166), which will further unlock the heterogeneity in complex diseases (Figure 1) (38, 111, 167). In the context of CVD, identifying the degree of heterogeneity of cell populations in complex tissues such as atherosclerotic plaques, calcified aortic valves, or cardiac muscle can lead to important insights into pathobiological mechanisms. For example, single-cell RNA sequencing has helped identify the diversity of the cardiac cellulome (168). Studies that combine network medicine and machine learning methods can be used to fine-tune our understanding and help identify a more accurate representation of the regulatory underpinnings of cellular heterogeneity (68, 103, 129–131, 147, 169). Moreover, the transfer of fundamental concepts across fields, such as a newly proposed statistical mechanics framework for single-cell biology (170), can further accelerate advances in systems medicine. As discussed, the integration of multiscale omics data may also facilitate the development of precision therapies. Finally, recent advances in harmonizing network biology methodologies with the power of machine learning (171–173) will soon come to full fruition, as high-resolution molecular data are increasingly converging with corresponding clinical and EHR data from individuals.

## Innovative technologies for driving precision cardiovascular medicine

### Proteomics headlines the multiomics universe of CVD research

**FIGURE 4:**
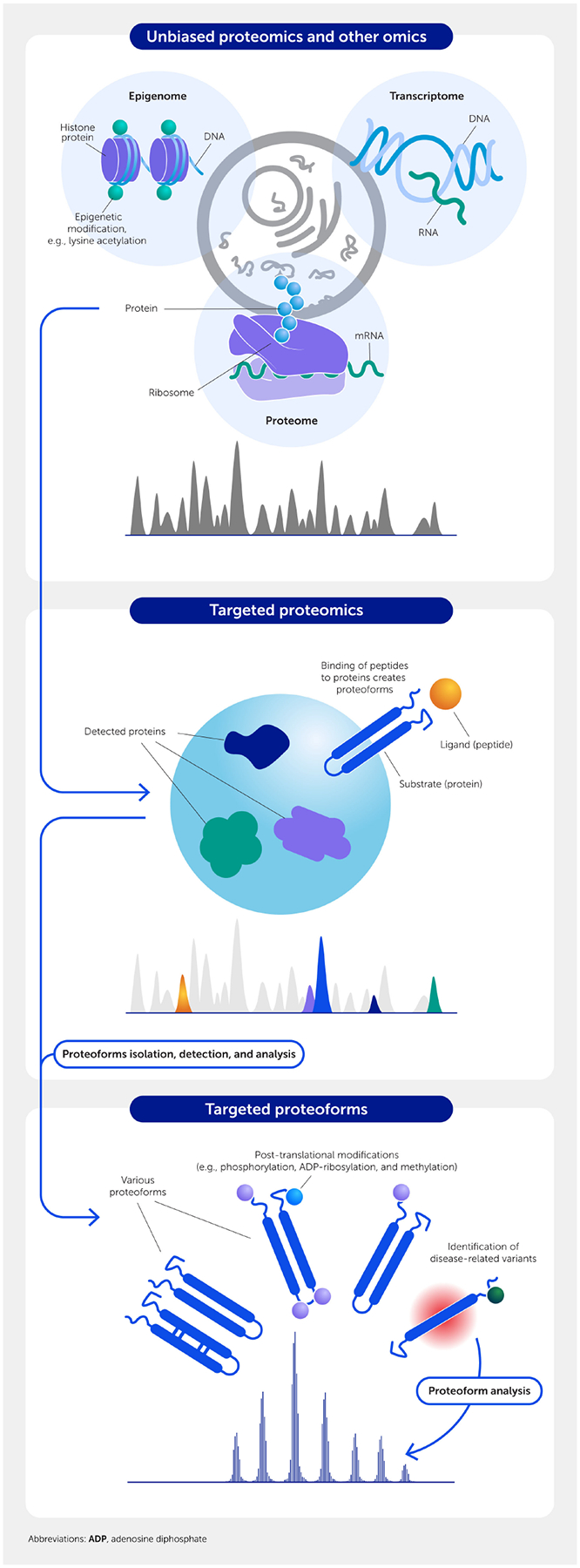
Drug-target discovery depends on multiple omics strategies. Unbiased screening of the epigenome, transcriptome, and/or proteome identifies candidate targets that can be validated using targeted mass spectrometry/proteomics. Ultimately, a specific proteoform (post-translationally modified form) of the protein may be the best target in some cases.

Omics technologies, which offer an unbiased survey of multiple genes and proteins, have significantly increased the likelihood of identifying potential therapeutic targets. This process can be effectively complemented with targeted proteomics and the more recently defined proteoforms resulting from post-translational modifications. Such an approach can facilitate successful target discovery and clinical translation (Figure 4), as discussed later in this section.

The high demand for continued discovery of additional therapeutic targets has driven the development of global, unbiased platforms such as epigenomics, transcriptomics, proteomics, and metabolomics. In particular, mass spectrometry-enabled protein research has long been recognized as a promising means to identify novel biomarkers and therapeutic targets for CVD (174–176). Today, mass spectrometry is a mainstay not only for proteome profiling (177) but also metabolome profiling (178, 179). When combined with other omics approaches, such as epigenomics (179) or transcriptomics (180), it provides solid foundations for systems biology and multiomics data integration strategies (Figures 1–4) (38, 167, 181).

Proteins are one of the major determinants of the cellular phenotype, driving initiatives such as the Human Proteome Project to facilitate translational research to improve overall human health (182). Unbiased proteomic approaches are consistently used to identify molecular drivers of CVDs, such as coronary heart disease (183), abdominal aortic aneurysms (148), and calcific aortic valve disease (129). In the last example, transcriptomics and proteomics were used to distinguish fibrotic and calcific regions from non-diseased regions of aortic valve leaflets but reported only a weak correlation between the quantified transcripts and proteins (129). These findings emphasize that protein abundances may not necessarily occur in proportion to their transcript abundances (184, 185). Moreover, tissues comprise various cell types such that “bulk RNA and proteome” data provide average signals, thereby eliminating the opportunity to glean potential “disease–driver subpopulations.” While single-cell transcriptomic technologies have been successfully implemented to reveal the extent of cellular subpopulations and heterogeneity in tissues (144), single-cell proteomics (186, 187) is still too recent a technology, requiring extensive expertise to yield a similar widespread implementation.

Targeted proteomics is already valued in a clinical setting, namely, to monitor steady-state kinetics of candidate LDL-C lowering targets such as apolipoprotein B (APOB), cholesteryl-ester transfer protein (CETP), and PCSK9 in cardiovascular outcome trials (188–190). Innovations in targeted mass spectrometry technologies enabled tracer kinetics studies in humans that captured the complex metabolic profiles of several high-density lipoprotein (HDL)-associated proteins, supporting the notion that HDL is a heterogeneous lipoprotein class consistent with its multiple functions (191, 192). These findings underscore that effective CVD drugs may require targeting a subpopulation with distinct functions rather than the entirety of a given molecule or cell class.

Proteins themselves comprise various isoforms—known as proteoforms—that may result from genetic variants, messenger RNA (mRNA) splice variants, and post-translational modifications, of which only one form may be causal to the disease of interest. Therefore, the proteomics community has initiated the Human Proteoform Project, an ambitious endeavor to generate a reference set of proteoforms for the human genome (193). Although mass spectrometry is a central technology supporting this initiative, it is, in essence, a multiomics endeavor (Figure 4) (193).

As first predicted over 20 years ago, mass spectrometry-enabled proteomics is providing CVD researchers multiple avenues through which to identify therapeutic targets. In all likelihood, the next CVD breakthrough targets may be identified using omics. Before its arrival on the market, however, the methodology will require extensive validation studies that, in part, may very well entail one or more additional proteomic technologies.

### Single-cell technologies to tackle the complex biology of cellular heterogeneity

**FIGURE 5:**
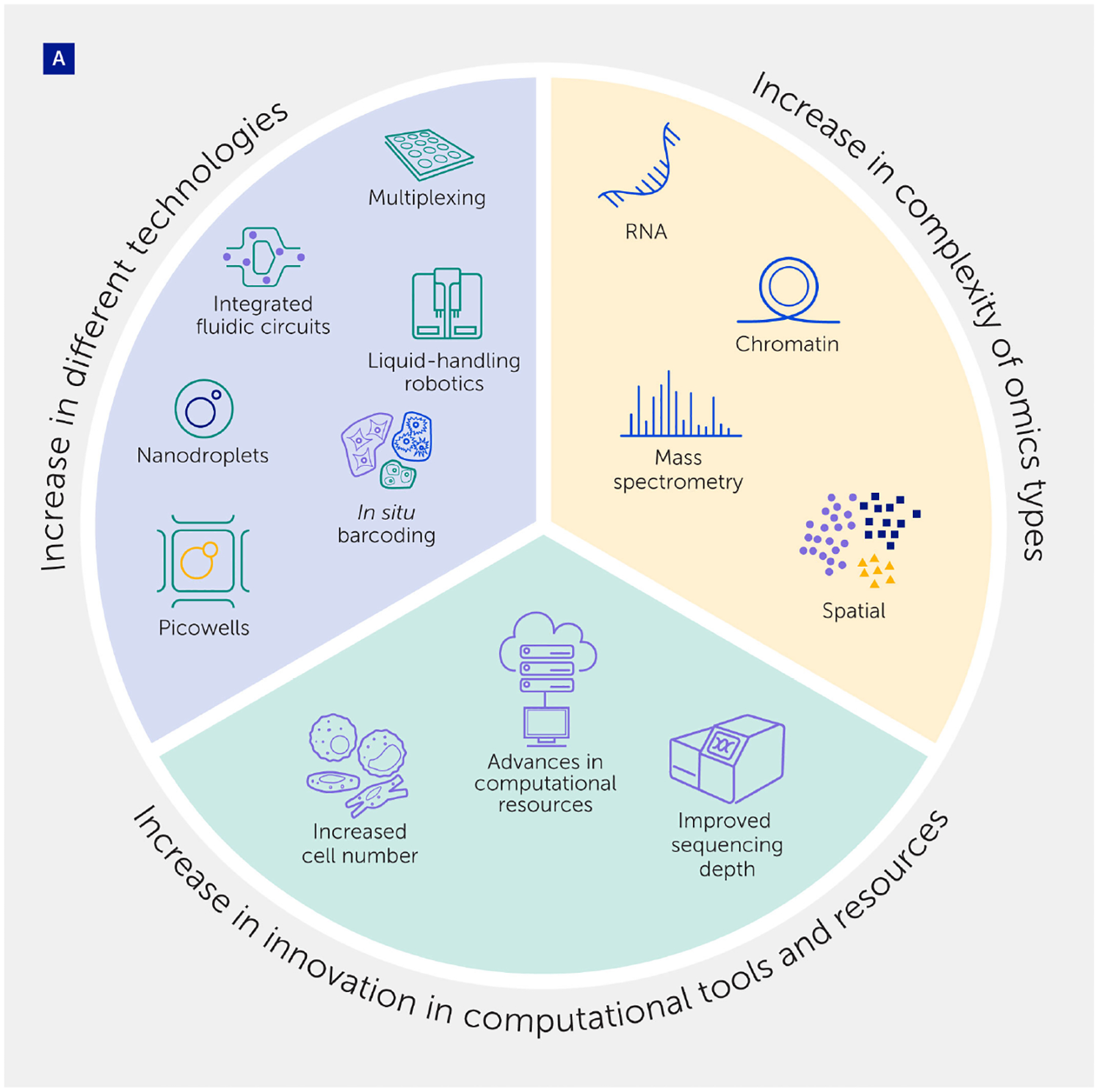

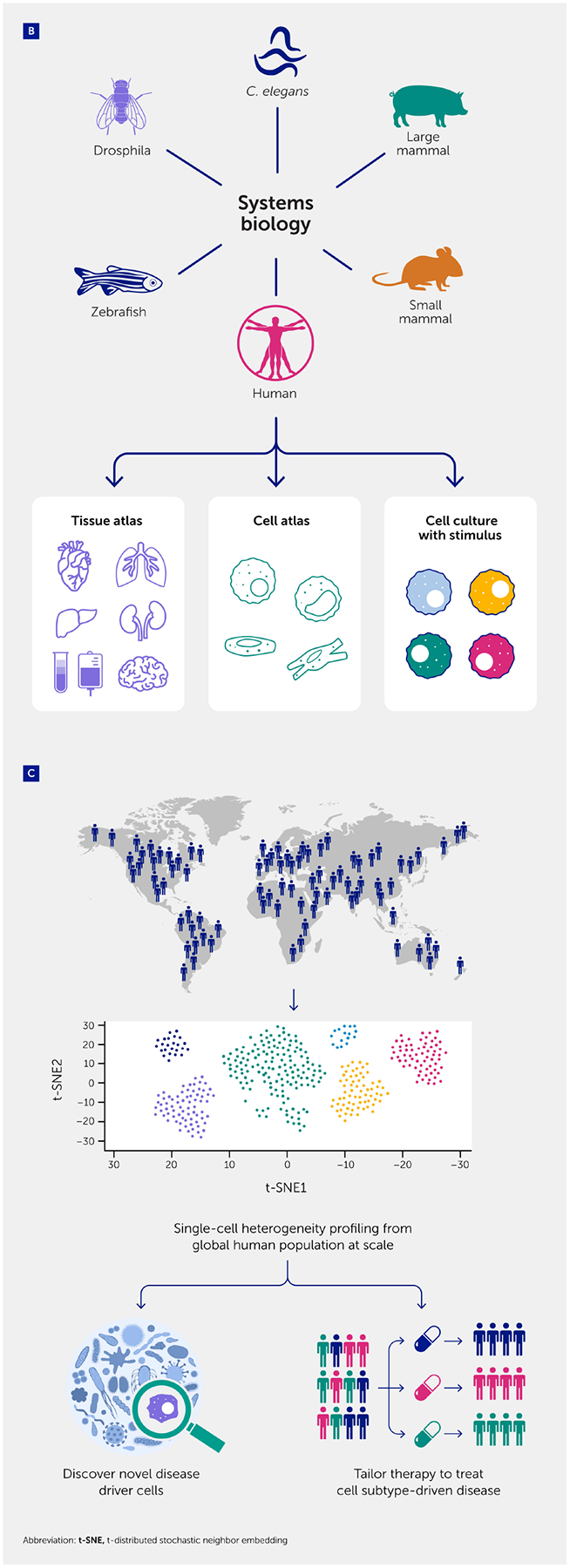
Integration of single-cell analyses for precision medicine. **(A)** An array of technological advances have occurred in platforms used for single-cell analysis, together with advances that have increased the complexity of omics types analyzed, and innovations in computational tools and resources. **(B)** Systems biology studies in animals and humans are now translating these advances into multi-tissue, single-cell atlases to provide *in vivo* landscapes of cellular heterogeneity. **(C)** Further extension of single-cell technologies to profile populations at scale could shape the future of biomedical research, establishing innovative diagnostics/ biomarkers and tailored therapies for diseases driven by specific cell subtypes.

As discussed, disease heterogeneity is a major challenge in cardiovascular medicine. For example, statins reduce the risk of acute complications such as myocardial infarction in many, but not all, patients. This can be attributed to factors such as the potency and pharmacodynamics of different statins, as well as patient heterogeneity. This may also result from different patterns of heterogeneity of atherosclerosis-associated cells (e.g., macrophages) among patients. Evidence has linked sustained pro-inflammatory activation of macrophages with vascular disorders (67, 194–196). An earlier paradigm of macrophage heterogeneity proposed a pro-inflammatory M1 phenotype and an anti-inflammatory/pro-resolving M2 phenotype (197, 198). More recent evidence (our own included) however, suggests that macrophage heterogeneity is more complex than the M1/M2 dichotomy, and involves more subpopulations (67, 199–202). While the overall balance of macrophage subpopulations may regulate disease mechanisms or severity, traditional assays only examine average levels of gene or protein expression in the entire population of cells (e.g., Western blot analysis and bulk RNA-sequencing) and cannot assess how individual cells behave. This challenge has driven the development of various platforms for single-cell analysis and their integration (Figure 5).

This challenge has been the catalyst for rapid and expansive development of single-cell technologies that enable deeper sequencing in more cells, platforms for simultaneous surveying of various omics layers in cells, and computational and bioinformatics infrastructure for innovative data analyses (Figure 5A). Technologies such as droplet-based, well-based, and sequential barcoding platforms can be chosen based on the underlying application. Single-cell analysis has extended beyond profiling RNA expression levels at the single-cell level with the ability to map surface expression of receptors (203) and chromatin accessibility (204). While these omics datasets can either be individually mined, they can also be paired with mRNA expression (205) by these cells to provide truly integrated multiomics characterization (206). Furthermore, recent advances in single-cell proteomics (207, 208) powered through mass spectrometry allow unbiased characterization of the proteome at the single-cell level. Spatial transcriptomics and spatial proteomics have also become widely available platforms that provide critical information relating to the spatial disposition of cellular heterogeneity. Leveraging these technologies allows for the construction of multilevel spatial multiomics maps through disease progression within the heart to identify signaling pathways specific to different cell types (59, 130, 209, 210). Assays that characterize different types of omics at single-cell resolution allow for the simultaneous measurement of epigenetics, transcriptomics, and proteomics. These include single-cell assays for transposase-accessible chromatin (ATAC)-sequencing for chromatin accessibility, single-cell profiling of histone modifications (211), spatial transcriptomic profiling (e.g., Slide-seq) (212), and surface receptor profiling (e.g., cellular indexing of transcriptomes and epitopes sequencing; CITE-seq) (213). Recent developments have expanded even into the realm of single-cell metabolomics, which provides opportunities to evaluate substrates and metabolites within the same cell (214–217). Such approaches will significantly advance integrative single-cell omics research.

The wide range of available, free software packages that can be used to analyze these datasets has also removed barriers to entry for many researchers, enabling them to embrace these approaches to address their specific research needs. This transition has coincided with the decreasing cost of cloud computing and the secure computing capabilities provided by research institutions and private companies that allow rapid, cost-effective processing of these large datasets (Figure 5A).

The rapid utilization of single-cell RNA-sequencing technologies (218) has enabled the construction of a wide range of single-cell atlases (219). Multi-tissue cell atlases of various model organisms have provided an important understanding of the *in vivo* landscape of cellular heterogeneity (220–222). Human-centric atlases have mapped specific organs that have been instrumental in identifying the various cell types and subtypes that make up an organ (219, 223, 224) (Figure 5B). Specific cell atlases discern how the same cell identified in multiple tissues has different underlying transcriptional signaling (58, 62–64, 225–227). Furthermore, recent studies have deployed single-cell RNA-sequencing technology to identify novel cell subtypes that could be disease drivers within specific disease settings (130, 228). These studies help shed light on specific cellular signaling aspects that regulate cellular heterogeneity.

While unbiased single-cell omics datasets have become increasingly cost-effective, generating, annotating, and sharing them remain expensive and resource-intensive. Reproducibility and data access are important aspects of large omics datasets that have made major strides recently. Easy and accessible computational pipelines are now widely available (229, 230). Journal requirements that make detailed single-cell datasets publicly available also help extend their utility once generated, which will also facilitate comparisons of datasets to enhance reproducibility.

Importantly, using publicly available unbiased single-cell omics data, researchers can fine-tune their approaches and generate follow-up experiments using targeted approaches to either validate these findings in a larger dataset or perform hypothesis-testing experiments. In this regard, the development of methods to perform targeted single-cell mRNA sequencing significantly reduces the costs of sequencing and facilitates the sequencing of a large number of cells (231). Similarly, the utilization of high-parameter flow cytometry (232) as well as sequential staining of tissue sections are becoming increasingly attractive (232, 233). Large-scale panels of validated probes and antibodies allow for the rapid adoption of these platforms across a wide range of tissues.

The characterization of cellular heterogeneity through these approaches helps to identify key cell types within *in vivo* settings. However, future studies can also use both unbiased and targeted approaches within monoculture systems to evaluate cellular heterogeneity in response to classical stimuli (234–236). Past efforts have typically utilized bulk omics studies to identify heterogeneity in responses (94) but are not limited in their capacity to highlight how different cells within a monoculture system can respond differently to the same stimulus (Figure 5B). Single-cell RNA sequencing and single-cell ATAC sequencing (237, 238) will allow the identification of novel subpopulations within a single cell type.

While single-cell technologies have developed rapidly, a few key questions remain (239). It is of critical importance to leverage cellular heterogeneity information to identify new mechanisms that translate into the clinic. We also need to consider the contribution of a small subset of disease driver cells to disease progression. Another point of consideration is the temporal dynamics of measured proteins and genes and their relative contribution to chronic disease. Most importantly, we also need to ask how understanding macrophage heterogeneity can provide molecular bases for the development of new diagnostics and therapies (240, 241). Recent studies offer examples of how cell heterogeneity data can be translated into drug development (169). How can we associate the information of subsets of cells associated with CVD with high-risk patients? Further extension of single-cell technologies, such as high-content live cell tracking for longitudinal monitoring and histologic localization of high-dimensional single-cell data in disease tissues, may help to facilitate clinical translation (31, 242–245). The combined use of single-cell data and computational drug screening methods (68, 169), discussed below, may also lead to potential new therapies. These methods may help us develop new approaches in precision cardiovascular medicine (246), establish innovative diagnostics/biomarkers (247), and enable intelligent enrollment criteria trial design, focused data interpretation, and improved patient safety in clinical trials (248, 249) (Figure 5C).

### AI supporting translational discoveries for complex CVD

Medical scientists have faced challenges in analyzing the massive biological and clinical datasets necessary to address the complexity and heterogeneity of human diseases. Exponential technological advances and their integration into basic science and clinical activities, such as omics and EHRs, have accelerated this trend. Over the last several decades, the field of AI has led to a major technological revolution that has already significantly impacted practically every aspect of the human experience, including medical research and practice (250, 251). However, the use of AI-powered technologies in medical sciences is not necessarily new. In the 1980s and 1990s, decision support systems, such as Health Evaluation through Logical Processing (HELP) or DXplain, assisted physicians through the diagnostic process (252, 253). However, with the increase in computational power and the availability of large volumes of data, AI has unveiled its extensive capabilities in the last decade.

**FIGURE 6:**
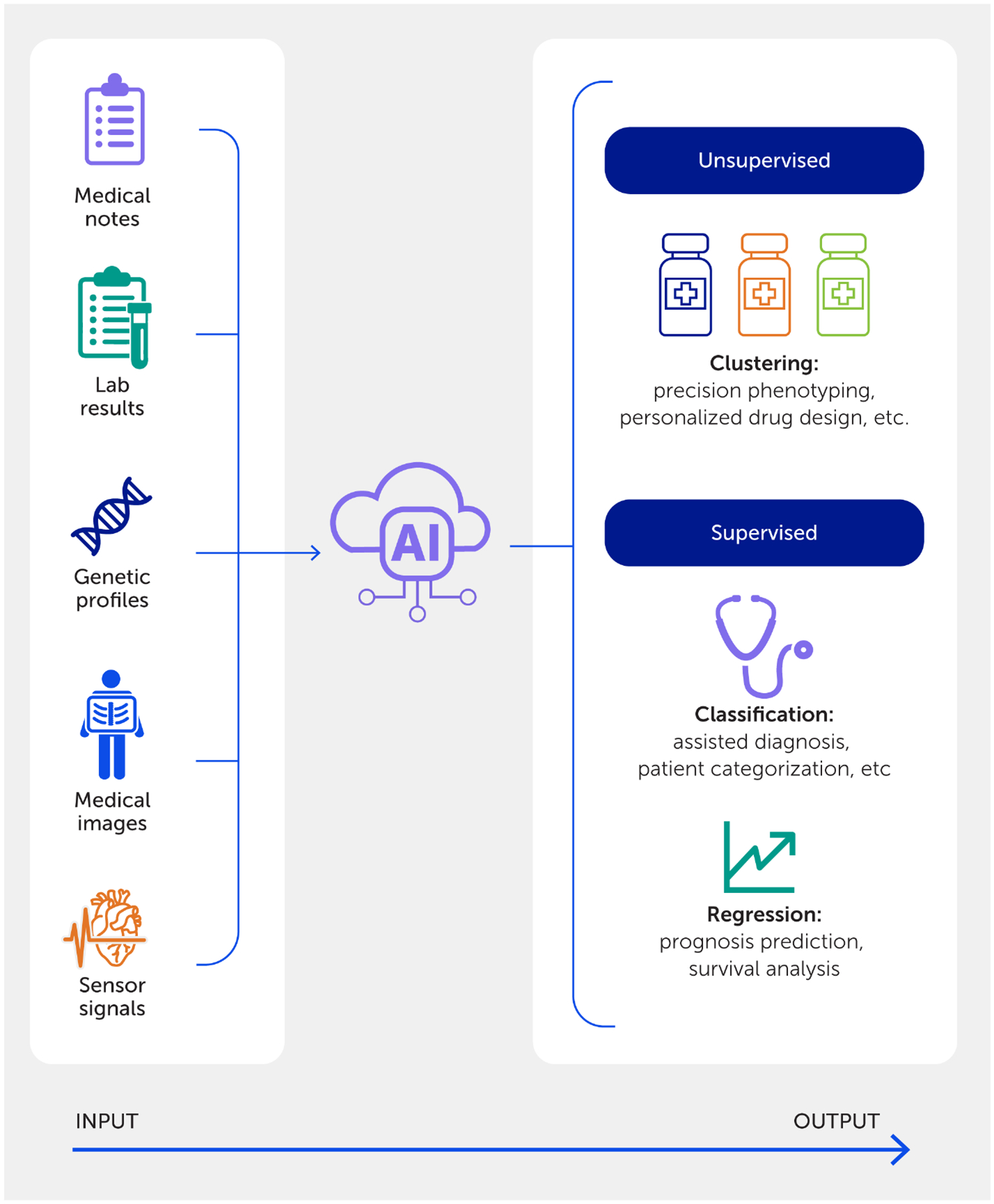
An artificial intelligence (AI)-powered precision medicine approach. An in-depth exploration of the AI workflow in medicine, highlighting diverse data inputs and delineating between supervised and unsupervised learning applications in clinical and research settings.

The application of AI in cardiovascular sciences has focused on two main tasks: prediction and clustering (Figure 6) (254–256). Prediction tools are used to estimate future prognosis and survivability of CVDs, including heart failure (257–259) and cardiomyopathies (260–263). AI prediction has also been implemented successfully to assist diagnosis (264–266), especially by using medical imaging data (e.g., echocardiogram, computed tomography, or magnetic resonance imaging) (267–274). Although not as common as contemporary AI-based prediction technologies, unsupervised learning has been used to cluster patient populations into different phenotypes (275–278), aiding the design of more precise therapeutic paths. Further, a new AI tool called AlphaMissense, which builds on the protein structure prediction tool AlphaFold2 (279), can be used to evaluate specific genetic variations (e.g., rare missense variations)—addressing the previous “bottleneck” in the bioinformatic analysis and assignment of causality to link a particular candidate genetic variant to the phenotype.

Considering differences among individuals, recent target discovery efforts have used large clinical data sources such as gene expression datasets associated with specific diseases and EHRs in addition to, or in place of, preclinical samples from cultured cells or animal models to address the biology of disease heterogeneity at the population level (71). The 21st Century Cures Act, initiated by the United States Government in 2016, promoted the use of clinical data sources such as the EHR (“real-world data”) in drug development and regulatory decision-making (280). Generative AI programs can autonomously create new content by learning patterns from existing data. AI’s role in changing the regulatory paradigms will involve improvements at all stages, from creating regulatory documents and designing the protocols to patient and site matching. The ability of AI to navigate huge datasets and construct detailed patient profiles based on demographics, medical history, and genetics to create “digital twins” can be used to simulate outcomes using virtual trials. These are the new AI frontiers: promises of refined therapeutics, improved patient care, and enhanced regulatory processes.

In the near future, integrating health records, clinical medical knowledge, and data provided by “smart” devices, such as phones and watches with AI technologies, will lead to unprecedented changes in our understanding of cardiovascular medicine (281). Technology companies such as IBM (282–284), Microsoft (285), Google (286), and Apple (287) have recognized its potential and have announced significant investments accordingly. The use of AI in continuous real-time monitoring, precision drug design, precision phenotyping, and the precise prediction of the development of CVDs represents a major breakthrough driven by new technology in the history of medicine.

### Data science-powered drug development

The cost and time required for drug discovery have increased annually, presenting the pharmaceutical industry with major challenges in developing and marketing new drugs (82–85, 288). Another challenge is the low success rate of new targets progressing to clinical stages and achieving favorable outcomes in clinical trials (88, 89, 91). As discussed earlier, generating compounds for undruggable targets is a major obstacle in the development of innovative drugs (99, 100). On the other hand, valuable medical, biological, and chemical data have accumulated, and the performance of computers in handling “big data” has evolved. To save cost and time, computational approaches have increasingly contributed to various aspects of drug discovery. In particular, the computational exploration and design of effective therapeutic compounds are major emerging fields. Such innovative technologies may also help to make traditionally undruggable targets druggable (101).

One such technique uses the quantitative structure–property relationship (QSPR) and quantitative structure–activity relationship (QSAR)—a prevalent statistical approach that correlates molecular structure with properties or biological activity using quantifiable descriptors. These descriptors are often generated through density functional theory (DFT) (289), a widely applied quantum theory to calculate the electronic structures of atoms and molecules. Selecting the most relevant descriptors among them poses a significant challenge, as they encapsulate molecular characteristics responsible for the observed biological activity or chemical properties (290). Recent advancements have introduced novel QSAR methodologies that enrich the analysis of bioactivity. Nevertheless, QSAR models require rigorous testing and validation to assess their predictive accuracy and practical applicability (291).

One resource for drug identification is the Connectivity Map (CMap) (292). This database includes changes in many gene expression profiles (“signatures”) that occur when various compounds are exposed to various cell types. The CMap has been expanded to include over 1 million signatures using over 20,000 small molecules through the introduction of the L1000 assay, a low-cost, high-throughput, and highly reproducible gene expression profiling method (293, 294). The L1000-based CMap quickly identifies small molecules that modify gene expression signatures by either reversing or mimicking the changes caused by certain diseases. Therefore, such approaches have been widely used for rapid drug repurposing (102, 103, 295–297). This phenotypic screening as a counterstrategy to traditional target-based drug discovery has been successful in the development of “first-in-class” drugs (298, 299).

In target-based drug discovery, which generally favors the development of “best-in-class” drugs, the identification of compounds that interact with target proteins is a key task (300, 301). Drug–target interactions (DTIs) have been experimentally surveyed using high-throughput screening. However, the number of compounds that can be tested this way is limited compared with the theoretical number of drug-like compounds—estimated to range from 10^23^ to 10^60^ (302). It is therefore desirable to narrow-down candidate compounds using computational approaches. Computational DTI prediction can be divided into ligand-based, docking-based, and chemogenomic approaches.

Ligand-based approaches exploit the principle that compounds structurally similar to a known binder of a target protein are likely to interact with that protein in a similar manner. While such methods are rational and easy to follow, nothing can be predicted when there is no compound known to bind the target protein.

Docking-based approaches calculate the binding affinities between compounds and target proteins by simulating their 3D structures. Although this approach can evaluate interactions with any compounds, it requires knowledge of the *in vivo* structure of the target protein. The prediction thus becomes more difficult for compounds that interact with membrane or receptor proteins owing to their complex, flexible structures.

Chemogenomic approaches utilize the compound’s physicochemical features, such as molecular fingerprints, and the protein’s genomic features, such as amino acid sequences. Machine learning models learn the pattern of these features required for the interactions by using known DTI datasets, and then the model predicts whether an unknown compound indeed interacts with the target protein. This approach has attracted attention recently because it overcomes the inherent disadvantages of the ligand-based and docking-based approaches (303). Various frameworks employing classical machine learning methods, such as support vector machine or random forest approaches, and advanced techniques, such as deep learning (DL), have been proposed as relevant computational models and have already improved DTI prediction accuracy significantly (304–306).

Scientists have also attempted to computationally design novel compounds with desired molecular profiles (e.g., bioactivity, drug metabolism, pharmacokinetics, or synthetic accessibility). In this field, denoted *de novo* molecular design, various generative models based on DL architecture, such as the recurrent neural network, variational autoencoder, and generative adversarial network models, have emerged (307–309). Benefiting from the remarkable development of AI, these AI-powered generative models create feasible, plausible, yet entirely new compounds that have never been synthesized in the real world. These generated compounds can be used seamlessly as a new compound library for DTI prediction. Moreover, by providing the L1000-based CMap signatures to the aforementioned generative models, the design of novel compounds that induce desired gene expression signatures has been used in an attempt to expand the applicable range of L1000-based CMap (310, 311).

Notably, computational approaches are beginning to be used to predict the tertiary structure of proteins. A recently developed AI-based algorithm, AlphaFold2, predicts 3D protein structures from the amino acid sequences with high accuracy (312). AlphaFold2 may accelerate DTI prediction, especially via docking-based and chemogenomic approaches, because it can provide accurate protein structures whenever an experimental protein is unavailable and extract more structural features than the amino acid sequences can when used alone (313). Through such improvements, computational approaches will likely have an increasingly important role in compound exploration for drug discovery.

### New therapeutic platforms: from proteins to RNAs

Most CVDs are currently treated with small-molecule drugs that are orally administered to bind to proteins contributing to disease mechanisms. As we discussed, however, some new targets are undruggable with conventional strategies. Therapeutic options other than small molecules include monoclonal antibodies against proteins, e.g., evolocumab and alirocumab targeting PCSK9 for familial hypercholesterolemia (314). While these types of drugs are effective and can overcome some druggability issues, targets are limited to cell membrane proteins or circulating proteins, and their production costs are high. Innovative technologies that enable targeting of undruggable targets include targeted protein degradation, such as proteolysis-targeting chimera (PROTAC) molecules that can degrade a target protein by controlling the ubiquitin–proteasome system. Compared with small molecules that block protein function but leave protein levels unchanged, small interfering RNA (siRNA) and PROTAC-based approaches can help modulate protein levels directly. While PROTAC technology has mainly been used for cancer targets, recent advances have extended its application to non-cancer diseases, particularly immune, inflammatory, and neurological disorders (315).

RNA-targeted interventions, a new class of innovative therapeutics, may overcome some of the aforementioned challenges (84, 104–106). Their advantages include (i) each gene of interest is potentially targetable by RNA therapeutics, whereas protein-targeted small molecules or antibodies can target only 0.05% of the human genome (316); (ii) manufacturing costs are lower than those of protein-targeted therapeutics; and (iii) development times are substantially shorter than those for conventional medicines. RNA interventions include antisense oligonucleotide (ASO), siRNA, clustered regularly interspaced short palindromic repeats (CRISPR)-based genome editing, aptamer, and mRNA vaccines (317–321).

The first ASO drug was fomivirsen, approved by the United States FDA in the late 1990s for the treatment of cytomegalovirus (CMV) retinitis (322, 323). Mipomersen, an ASO targeting apolipoprotein-B-100 mRNA, was the first RNA-targeted therapy approved by the FDA for a CVD—familial hypercholesterolemia (324). Inclisiran, an siRNA targeting PCSK9, has proved safe and effective for lowering LDL (by approximately 50%) and cardiovascular outcome trials are ongoing (325). Another developmental ASO, pelacarsen, is directed against lipoprotein(a) [Lp(a)], which is linked clinically with CVD, including aortic stenosis. Specifically, pelacarsen targets the production of apolipoprotein(a) [Apo(a)], a key component of Lp(a) disulfide-linked to apolipoprotein B100. Pelacarsen proved safe and lowered Lp(a) levels by up to 80% in phase 2 trials (326). Clinical trials of siRNAs that reduce both normal and mutated transthyretin (TTR), causing TTR amyloidosis, reported attenuated progression not only of the associated peripheral neuropathy but also cardiomyopathy (327, 328). In addition to these developments, the COVID-19 mRNA vaccines showed that delivering native or chemically modified (e.g., pseudo-uridine) mRNA by encapsulation in lipid nanoparticles is another potential option to treat various diseases, including CVD. Yet, all such methods are directed toward interacting with proteins.

We know from increased usage of next-generation sequencers that most of our coding genome is transcribed as RNA (329). Only a small percentage codes for proteins, leaving a majority of transcribed RNAs as non-protein-coding RNAs (ncRNAs). In addition to the well-known ncRNAs, ribosomal RNAs (rRNAs), and transfer RNAs (tRNAs), other regulatory ncRNAs have been identified and characterized in recent years, including microRNAs (miRNAs), circular RNAs, and long ncRNAs (lncRNAs) (330). Not surprisingly, the dysregulation of ncRNAs is linked to various CVD etiologies, and hence, these ncRNAs are being investigated as potential CVD diagnostic biomarkers or therapeutic targets (331).

Preclinical and clinical trials of miRNA-based therapeutics for CVDs are ongoing (332) while most projects on lncRNA-targeted therapeutics are still in the preclinical stage. LncRNAs are associated with many human diseases and many efforts are underway to develop technologies to target them therapeutically (333, 334). LncRNAs involve diverse modes of action, providing different opportunities to modify their functions (e.g., via siRNAs, ASOs, CRISPR/Cas9, small molecules). Some mitochondrial lncRNAs have reached clinical trials as cancer therapies (334). Accumulating preclinical evidence has implicated lncRNAs in the pathogenesis of various CVDs, including atherosclerosis, myocardial infarction, heart failure, and arrhythmias, providing molecular bases for their clinical applications as therapeutic targets or biomarkers (331, 335, 336). While lncRNA-targeted therapeutics have high potential, their clinical development is lagging. This may be due to our incomplete understanding of their mechanism of action, necessitating more mechanistic studies of each lncRNA. In addition, innovative computational methods should help elucidate their interactions with miRNAs, coding RNAs, and proteins. The combined use of such targeted systems approaches will help translate advances in lncRNA biology into clinical CVD medicines.

These novel modalities can also be partnered with a wide array of drug delivery strategies to maximize their effectiveness (337) and reduce off-target effects (338). These have been key partnerships for emerging modalities, such as RNA-targeted therapeutics (339). While these drug delivery methods remain unproven in the clinic, they have accelerated preclinical research by serving as powerful tools for *in vivo* intervention (95, 144).

## Transforming cardiovascular medicine: innovative approaches and collaborative initiatives

### Interdisciplinary and multistakeholder drug discovery drives innovation

Facilitating drug discovery and development for innovative precision medicine requires new paradigms. New technological developments can help solve specific technical limitations and promote scientific discoveries. These discoveries can be accelerated by models that integrate multiple innovative technologies to holistically address the biology of heterogeneity, identify promising drug targets, predict their clinical impacts, and design, generate, and test new drugs. Dynamic and close collaboration between biologists and data scientists is essential to establish fully integrated drug discovery research, as discussed above. Such seamless approaches also require innovative cross-sector collaboration.

**FIGURE 7:**
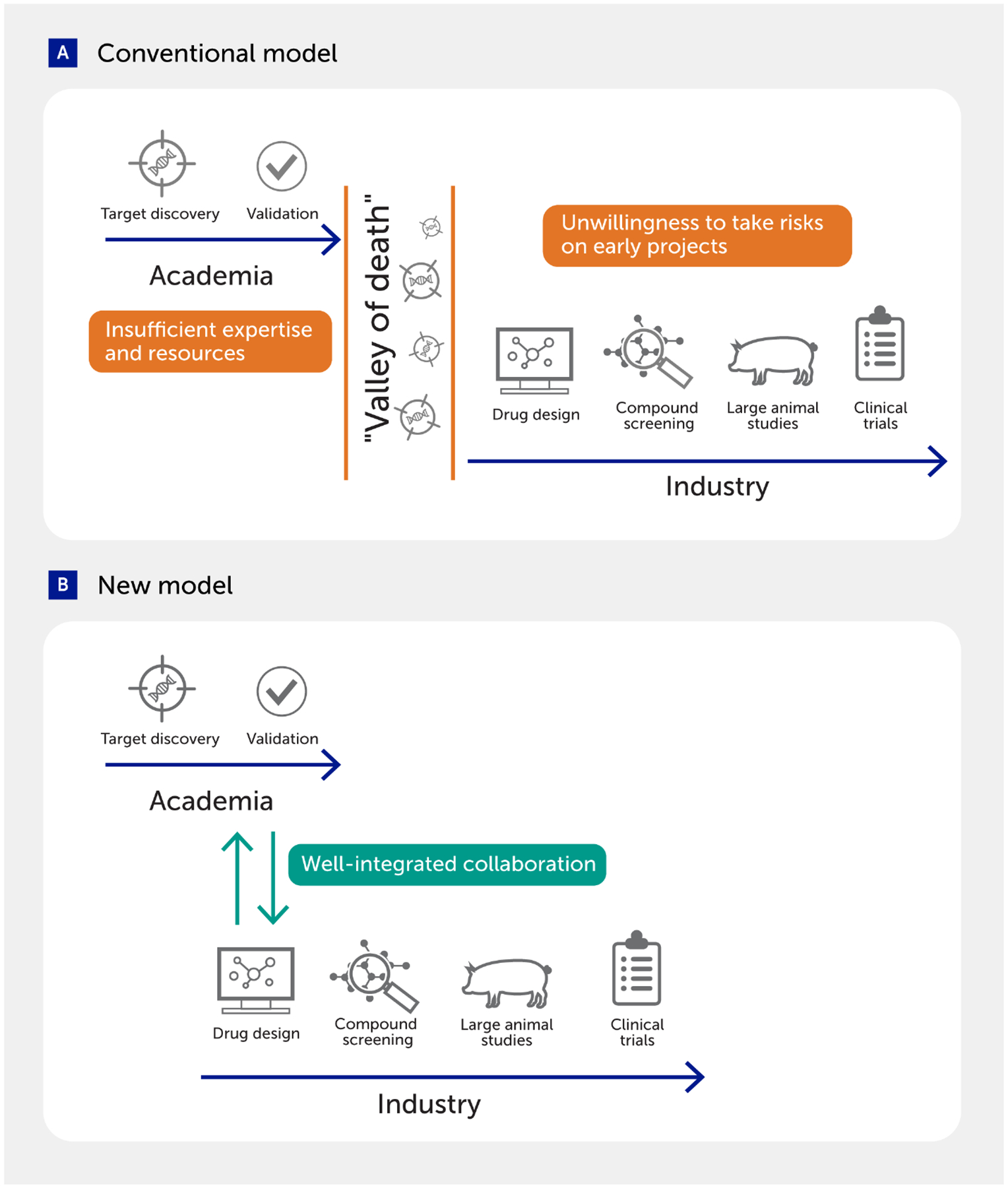
Academia–industry collaboration to fill the gap in drug discovery. **(A)** Several challenges often impair the transition of targets discovered in academia to drug development in industry. **(B)** New models that integrate actions in the two sectors may facilitate the translation of discoveries into the clinic.

One of the major obstacles is the large gap between target discovery research in academia and drug development in industry (Figure 7). Many ideas or targets identified in academic research do not bridge this gap for various reasons, including the lack of expertise and funding in academia and industry’s unwillingness to invest in early, high-risk projects. To solve these major challenges, several models of academia–industry collaboration have been established to merge the strengths of both sectors (89, 340–344). Establishing novel concepts by exploring uncharted territories and pursuing high-risk projects is a typical strength of academic investigators, while industrial scientists have specific expertise in drug design and development and are more strongly supported by infrastructure and financial resources (341, 342). Indeed, one study indicated that academia–industry collaboration showed higher clinical development success rates than those commonly seen in either academia or industry with no collaboration (89). Other types of collaborative arrangements include precompetitive research between pharmaceutical companies for sharing resources and expertise, and public–private partnerships (345–348).

### Collaborative data science: key to precision medicine

As discussed, the major challenges in CVD have prompted precision medicine approaches that in turn necessitate new technologies; these disruptive innovations not only solve challenges, but also generate new concepts. The key, essential component in this synergistic relationship is data science (349). Among new technologies across various disciplines, the evolution of data science has been particularly rapid. It is now critical for us to recognize the importance of implementing this discipline and involving data scientists in every stage of cardiovascular medicine innovation, from basic science, discovery, and translational research to the clinical development of new therapeutics and ultimately their use in clinical practice. We must also recognize the diversity of data science as characterized by various subspecialties (e.g., biostatistics, bioinformatics, biophysics, network science, computational biology, and machine learning-based approaches), which enables the construction of a multidisciplinary data science team to cover a wide range of needs. More resources need to be allocated to support the development of data scientists at the institutional and government levels to bolster future biomedical innovation in both academia and industry. Finally, infrastructures that support interoperability between the multiple data sources involved are also vital to enhance the synergistic relationships between data science and cardiovascular medicine (350).

### Lessons learned from COVID-19: are we ready for the next pandemic?

During the COVID-19 pandemic, over 770 million people were infected with the SARS-CoV-2 virus globally, leading to 7 million deaths (351). The scientific community came together to respond to the rapidly evolving demands that arose as a result. Public, political, and scientific awareness enabled resources to be redirected toward combating this global threat. This also triggered research interests in investigating the extensive, long-term consequences of viral infection on a large scale (352).

The United States FDA demonstrated flexibility and innovation during this time by modifying existing regulations to accelerate the approval process of life-changing medications for COVID-19 (353, 354). Similarly, the World Health Organization (WHO) played a central role in generating and distributing guidelines and tools to the global medical community (https://covid19.who.int). As a result, novel technologies, including mRNA vaccines and neutralizing antibodies (355, 356), were deployed with unprecedented rapidity to help reduce disease severity. Although these groundbreaking tools had already been in development (357, 358), the COVID-19 pandemic created the impetus to embrace these platforms to supplement conventional treatments. This new attention toward acute viral infection required the scientific, medical, and regulatory communities to restructure and shorten the drug development and implementation timeline (359). Governmental programs, such as the National Institutes of Health RECOVER program in the United States (360) (https://recovercovid.org/) to characterize post-acute sequelae of SARS-CoV-2 infection (PASC) syndrome or “long COVID” promoted the formation of multi-institutional and multidisciplinary nationwide collaborations by eliminating barriers to interactive science (361). Technologies developed through this global effort will be applied to counter other diseases, such as cardiovascular, pulmonary, and neurological disorders.

Knowledge gained in social, political, and clinical realms has shaped how our scientific community responds to worldwide challenges. The advent of new technologies has heightened the level of responsibility, as they allow the evaluation of interventions more quickly, more precisely, and at greater scales than was previously possible (362, 363). It will be essential to capitalize on the collaborations established during this crisis to address future pandemics successfully.

### Revamping global healthcare policies to tackle the leading global cause of mortality

We have discussed that investing in innovative approaches and cutting-edge technologies will help develop treatments and interventions that improve CVD outcomes. However, we cannot afford to miss the forest for the trees. The “domino effect” of anthropogenic causes of mortality is the most pressing human health problem in modern times (364). Climate change leads to increased natural disasters, which, in turn, cause changes in food and water security. It also results in supply chain disruptions and population displacements. All of these consequences collectively strain the healthcare system. This strain exacerbates the impact of both lifestyle and environmental components on the development and progression of CVD. There is also a heightened risk of respiratory and vector-borne diseases due to these interconnected factors (365).

Concerted efforts applied to the population scale of the Millennium Development Goals and the Sustainable Development Goals (SDGs) are needed to increase public awareness of CVD and its risk factors (366, 367). Precision medicine approaches can be a huge asset to meet SDG 3.4, which aims to reduce premature mortality from non-communicable diseases through prevention and treatment. Prevention of these diseases should be emphasized by encouraging healthy lifestyles, tobacco cessation, and more nutritious dietary alternatives. Strengthening healthcare infrastructure, increasing equitable access to quality healthcare, reducing economic disparities (368), and promoting unanimous support to the WHO’s *Global Action Plan for the Prevention and Control of NCDs* (369, 370) are necessary to, in some measure, mitigate this so-called “disease of civilization” (371).

### Political imperative to ensure global equity

The COVID-19 pandemic exposed the vulnerabilities of global healthcare systems: the lack of cooperation between governments and the private sector, unequal distribution of medical resources (including vaccines), and the neglect of needs in the global south. In the context of CVD, neglect of the disease burden in low-income and lower-middle-income countries (LICs/LMICs) and excluding such populations from research (including clinical trials) has led to major healthcare crises (372). Even though the disease burden is high in LICs and LMICs, these countries’ contributions to the global research output are minimal in part due to limited research capacity (373). Moreover, at present, global responses to the major global health threats from both noncommunicable and infectious diseases are hampered by geopolitical division, conflict, and power imbalances. (374) Achieving global health equity requires multisectoral, multifaceted, and multistakeholder engagements (375) through the emergence of global frameworks such as the WHO pandemic agreement adopted in May 2025 (376). While pandemics are temporary, continued cooperation and dedication of financial resources toward research and outreach are critical to mitigate future calamities. Effective global leadership is contingent upon the constructive global healthcare discourse among all member states, alongside proactive contributions from intergovernmental institutions. For this to happen, major policy stakeholders must recognize their lack of attention to the CVD burden and the insufficient healthcare infrastructure of the Global South, consequently amplifying their struggles. Concerted efforts addressing the issues plaguing individuals living in LICs/LMICs will be globally beneficial by reducing the burden of communicable and non-communicable diseases alike. Ultimately, addressing the disparities in global healthcare is not just a moral imperative but also a strategic necessity in safeguarding the well-being of all humans. By fostering collaboration, empathy, and a shared commitment to collective welfare, we can build a world where access to healthcare is a fundamental right for every individual, regardless of geographic location or socioeconomic status.

**FIGURE 8:**
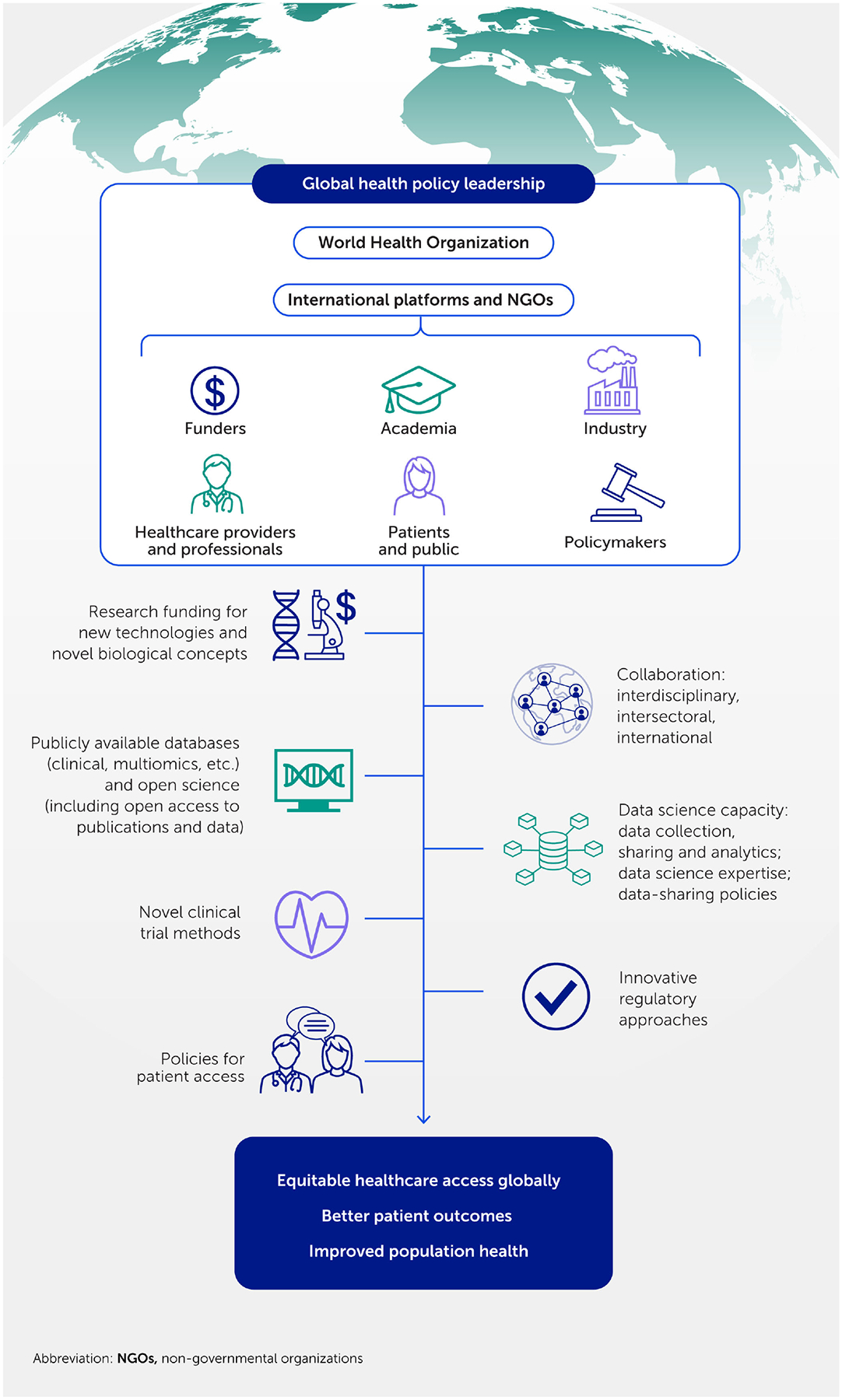
Global policy leadership fuels transformative cardiovascular innovations. Revolutionizing healthcare demands an unprecedented commitment from global policy leaders to foster interdisciplinary collaborations, funding, and support. This will drive innovation, enhance data science capacity, establish patient-centric public health policies, and concert efforts to reduce global inequality, addressing the silent pandemic of cardiovascular disease.

Therefore, transforming to a precision innovation paradigm in cardiovascular medicine will require more than the scientific and technological advances described above. Interdisciplinary, intersectoral and global collaborations throughout the research and innovation pathway (352, 377, 378), underpinned by global health policy leadership, are necessary to implement suitable models of research funding and organization, data infrastructures and policies, novel clinical trial methods, medicines regulation, and patient access policies (Figure 8).

## Conclusions

We have discussed how major challenges and needs in clinical cardiovascular medicine have provided opportunities for the scientific and medical communities to implement innovations and develop the systems approach needed to facilitate the search for disease mechanisms and establish unconventional strategies in drug development. The rapid evolution of cutting-edge technologies has recently increased our understanding of the biology of heterogeneity at cellular and patient levels, which would enable the establishment of new paradigms of precision medicine for CVDs. Advanced computational methods help to make traditionally undruggable targets druggable. New platforms, such as RNA therapeutics, facilitate modulation of undruggable targets (379).

Disruptive innovation not only solves challenges, but also leads us to new paradigms. Establishing new concepts in turn requires the development of new technologies. The key to successful cardiovascular innovation is a synergistic relationship between new technology and new paradigms, supported by a dynamic and intimate interplay between biomedical research and data science. In addition, cross-sector (e.g., academia–industry) or international partnerships can help to defend against global residual cardiovascular risks and address unmet medical needs. We saw this in action during the COVID-19 pandemic, where our community was forced to develop “borderless” solutions swiftly. This unprecedented challenge brought about a worldwide effort among scientists in academia and industry, leading to progress in comprehending virus transmission, effectively accelerating the development of novel technologies (e.g., mRNA vaccines), and reorganizing the medical community to improve responses to future crises. Such seamless collaboration across various disciplines, sectors, and nations will shift innovation paradigms to revolutionize borderless cardiovascular medicine and speed up the translation of discoveries into the clinic.

## Data Availability

The original contributions presented in this work are included in the article. Further inquiries can be directed to the corresponding author.

## Key points

- Heterogeneity in the complex pathobiology and presentation of cardiovascular diseases (CVDs)—the leading cause of death globally—limits the effectiveness of conventional “one-size-fits-all” therapies.
- Integrative systems approaches, which involve unbiased omics analyses (especially proteomics and single-cell analyses), bioinformatics, and network science, now offer the potential for a precision medicine innovation paradigm to tackle CVDs.
- Artificial intelligence (AI) is driving new opportunities in patient profiling and computational precision drug design and development.
- Various RNA therapeutics in development offer promise for effective precision therapy and could help address conventional drug development obstacles, such as cost and time.
- Interdisciplinary and intersectoral collaboration throughout the research and innovation pathway, underpinned by global health policy leadership, is necessary to implement a precision cardiovascular medicine paradigm.

## References

[1] Martin SS, Aday AW, Allen NB, Almarzooq ZI, Anderson CAM, Arora P, 2025 heart disease and stroke statistics: a report of US and global data from the American Heart Association. Circulation (2025) 151(8):e41–660. doi: 10.1161/CIR.0000000000001303 39866113 PMC12256702

[2] Mensah GA, Fuster V, Murray CJL, Roth GA, Global Burden of Cardiovascular Diseases and Risks Collaborators. Global burden of cardiovascular diseases and risks, 1990–2022. J Am Coll Cardiol (2023) 82(25):2350–473. doi: 10.1016/j.jacc.2023.11.007 38092509 PMC7615984

[3] Fuster V, Badimon L, Badimon JJ, Chesebro JH. The pathogenesis of coronary artery disease and the acute coronary syndromes. N Engl J Med (1992) 326(4):242–50. doi: 10.1056/NEJM199201233260406 1727977

[4] Libby P Molecular bases of the acute coronary syndromes. Circulation (1995) 91(11):2844–50. doi: 10.1161/01.cir.91.11.2844 7758192

[5] Fayad ZA, Robson PM, Fuster V. Rethinking heart attack prevention: the myth of the “vulnerable plaque” and reality of patient risk. J Am Coll Cardiol (2024) 83(22):2145–7. doi: 10.1016/j.jacc.2024.04.007 38811092

[6] Kraler S, Mueller C, Libby P, Bhatt DL. Acute coronary syndromes: mechanisms, challenges, and new opportunities. Eur Heart J (2025) 46(29):2866–89. doi: 10.1093/eurheartj/ehaf289 40358623 PMC12314746

[7] Groenewegen A, Rutten FH, Mosterd A, Hoes AW. Epidemiology of heart failure. Eur J Heart Fail (2020) 22(8):1342–56. doi: 10.1002/ejhf.1858 32483830 PMC7540043

[8] Rasalam R, Sindone A, Deed G, Audehm RG, Atherton JJ. State of precision medicine for heart failure with preserved ejection fraction in a new therapeutic age. ESC Heart Fail (2025) 12(3):1544–57. doi: 10.1002/ehf2.15205 39844745 PMC12055434

[9] Gupta P, Bast JA, Razavi AC, Canonico ME, Shahzad A, Naeem M, Hypertension in atherosclerotic cardiovascular disease: insights into epidemiology, management strategies, and outcomes. Curr Opin Cardiol (2025) 40(4):206–12. doi: 10.1097/HCO.0000000000001226 40305167

[10] Blaser MC, Bäck M, Lüscher TF, Aikawa E. Calcific aortic stenosis: omics-based target discovery and therapy development. Eur Heart J (2025) 46(7):620–34. doi: 10.1093/eurheartj/ehae829 39656785 PMC11825147

[11] Buja LM, Nikolai N. Anitschkow and the lipid hypothesis of atherosclerosis. Cardiovasc Pathol (2014) 23(3):183–4. doi: 10.1016/j.carpath.2013.12.004 24484612

[12] Steinberg D In celebration of the 100th anniversary of the lipid hypothesis of atherosclerosis. J Lipid Res (2013) 54(11):2946–9. doi: 10.1194/jlr.R043414 23975896 PMC3793599

[13] Osler W Angina pectoris and arteriosclerosis. JAMA (2015) 314(18):1981. doi: 10.1001/jama.2014.12084 26547477

[14] Classics in arteriosclerosis research: on experimental cholesterin steatosis and its significance in the origin of some pathological processes by N. Anitschkow and S. Chalatow, translated by Mary Z. Pelias, 1913. Arteriosclerosis (1983) 3(2):178–82 doi: 10.1161/01.ATV.3.2.178 6340651

[15] Mahmood SS, Levy D, Vasan RS, Wang TJ. The Framingham heart study and the epidemiology of cardiovascular disease: a historical perspective. Lancet (2014) 383(9921):999–1008. doi: 10.1016/S0140-6736(13)61752-3 24084292 PMC4159698

[16] Packard C, Chapman MJ, Sibartie M, Laufs U, Masana L. Intensive low-density lipoprotein cholesterol lowering in cardiovascular disease prevention: opportunities and challenges. Heart (2021) 107(17):1369–75. doi: 10.1136/heartjnl-2020-318760 33795379 PMC8374039

[17] Aday AW, Ridker PM. Targeting residual inflammatory risk: a shifting paradigm for atherosclerotic disease. Front Cardiovasc Med (2019) 6:16. doi: 10.3389/fcvm.2019.00016 30873416 PMC6403155

[18] Libby P, Aikawa M. Stabilization of atherosclerotic plaques: new mechanisms and clinical targets. Nat Med (2002) 8(11):1257–62. doi: 10.1038/nm1102-1257 12411953

[19] Corsini A, Ginsberg HN, Chapman MJ. Therapeutic PCSK9 targeting: inside versus outside the hepatocyte? Pharmacol Ther (2025) 268:108812. doi: 10.1016/j.pharmthera.2025.108812 39947256

[20] Tokgözoğlu L, Libby P. The dawn of a new era of targeted lipid-lowering therapies. Eur Heart J (2022) 43(34):3198–208. doi: 10.1093/eurheartj/ehab841 35051271 PMC9448630

[21] Dhindsa DS, Sandesara PB, Shapiro MD, Wong ND. The evolving understanding and approach to residual cardiovascular risk management. Front Cardiovasc Med (2020) 7:88. doi: 10.3389/fcvm.2020.00088 32478100 PMC7237700

[22] Patel KV, Pandey A, de Lemos JA. Conceptual framework for addressing residual atherosclerotic cardiovascular disease risk in the era of precision medicine. Circulation (2018) 137(24):2551–3. doi: 10.1161/CIRCULATIONAHA.118.035289 29643058

[23] Cesaro A, De Michele G, Fimiani F, Acerbo V, Scherillo G, Signore G, Visceral adipose tissue and residual cardiovascular risk: a pathological link and new therapeutic options. Front Cardiovasc Med (2023) 10:1187735. doi: 10.3389/fcvm.2023.1187735 37576108 PMC10421666

[24] Skrobucha A, Pindlowski P, Krajewska N, Grabowski M, Jonik S. Anti-inflammatory effects of glucagon-like peptide-1 (GLP-1) in coronary artery disease: a comprehensive review. Front Cardiovasc Med (2024) 11:1446468. doi: 10.3389/fcvm.2024.1446468 39741663 PMC11685754

[25] Pratley RE, Tuttle KR, Rossing P, Rasmussen S, Perkovic V, Nielsen OW, Effects of semaglutide on heart failure outcomes in diabetes and chronic kidney disease in the FLOW trial. J Am Coll Cardiol (2024) 84(17):1615–28. doi: 10.1016/j.jacc.2024.08.004 39217553

[26] Garofolo M, Penno G, Solini A, Orsi E, Vitale M, Resi V, Relationship between degree of risk factor control and all-cause mortality in individuals with type 2 diabetes: a prospective cohort study. Eur J Intern Med (2024) 128:53–62. doi: 10.1016/j.ejim.2024.05.034 38845288

[27] Vaughan AS, Coronado F, Casper M, Loustalot F, Wright JS. County-level trends in hypertension-related cardiovascular disease mortality-United States, 2000 to 2019. J Am Heart Assoc (2022) 11(7):e024785. doi: 10.1161/JAHA.121.024785 35301870 PMC9075476

[28] Tsao CW, Aday AW, Almarzooq ZI, Alonso A, Beaton AZ, Bittencourt MS, Heart disease and stroke Statistics-2022 update: a report from the American Heart Association. Circulation (2022) 145(8):e153–639. doi: 10.1161/CIR.0000000000001052 35078371

[29] Benjamin EJ, Blaha MJ, Chiuve SE, Cushman M, Das SR, Deo R, Heart disease and stroke Statistics—2017 update: a report from the American Heart Association. Circulation (2017) 135(10):e146–603. doi: 10.1161/CIR.0000000000000485 28122885 PMC5408160

[30] Roth GA, Mensah GA, Johnson CO, Addolorato G, Ammirati E, Baddour LM, Global burden of cardiovascular diseases and risk factors, 1990–2019: update from the GBD 2019 study. J Am Coll Cardiol (2020) 76(25):2982–3021. doi: 10.1016/j.jacc.2020.11.010 33309175 PMC7755038

[31] Chelvanambi S, Decano JL, Winkels H, Giannarelli C, Aikawa M. Decoding macrophage heterogeneity to unravel vascular inflammation as a path to precision medicine. Arterioscler Thromb Vasc Biol (2024) 44(11):2253–7. doi: 10.1161/ATVBAHA.124.319571 39441912 PMC11715277

[32] Smith MT, de la Rosa R, Daniels SI. Using exposomics to assess cumulative risks and promote health. Environ Mol Mutagen (2015) 56(9):715–23. doi: 10.1002/em.21985 26475350 PMC4636923

[33] Vineis P, Robinson O, Chadeau-Hyam M, Dehghan A, Mudway I, Dagnino S. What is new in the exposome? Environ Int (2020) 143:105887. doi: 10.1016/j.envint.2020.105887 32619912

[34] Bucher ML, Anderson FL, Lai Y, Dicent J, Miller GW, Zota AR. Exposomics as a tool to investigate differences in health and disease by sex and gender. Exposome (2023) 3(1):osad003. doi: 10.1093/exposome/osad003 PMC10125831 37122372

[35] Baptiste DL, Turkson-Ocran RA, Ogungbe O, Koirala B, Francis L, Spaulding EM, Heterogeneity in cardiovascular disease risk factor prevalence among white, African American, African immigrant, and Afro-Caribbean adults: insights from the 2010–2018 national health interview survey. J Am Heart Assoc (2022) 11(18):e025235. doi: 10.1161/JAHA.122.025235 36073627 PMC9683685

[36] Rajagopalan S, Landrigan PJ. Pollution and the heart. N Engl J Med (2021) 385(20):1881–92. doi: 10.1056/NEJMra2030281 34758254

[37] Zhang A, Wu Z, Wu E, Wu M, Snyder MP, Zou J, Leveraging physiology and artificial intelligence to deliver advancements in health care. Physiol Rev (2023) 103(4):2423–50. doi: 10.1152/physrev.00033.2022 37104717 PMC10390055

[38] Sonawane AR, Aikawa E, Aikawa M. Connections for matters of the heart: network medicine in cardiovascular diseases. Front Cardiovasc Med (2022) 9:873582. doi: 10.3389/fcvm.2022.873582 35665246 PMC9160390

[39] Chandy M, Obal D, Wu JC. Elucidating effects of environmental exposure using human-induced pluripotent stem cell disease modeling. EMBO Mol Med (2022) 14(11): e13260. doi: 10.15252/emmm.202013260 36285490 PMC9641419

[40] Kathiresan S, Srivastava D. Genetics of human cardiovascular disease. Cell (2012) 148(6):1242–57. doi: 10.1016/j.cell.2012.03.001 22424232 PMC3319439

[41] Soremekun O, Dib MJ, Rajasundaram S, Fatumo S, Gill D. Genetic heterogeneity in cardiovascular disease across ancestries: insights for mechanisms and therapeutic intervention. Camb Prism Precis Med (2023) 1:e8. doi: 10.1017/pcm.2022.13 38550935 PMC10953756

[42] Gurdasani D, Barroso I, Zeggini E, Sandhu MS. Genomics of disease risk in globally diverse populations. Nat Rev Genet (2019) 20(9):520–35. doi: 10.1038/s41576-019-0144-0 31235872

[43] Woodward AA, Urbanowicz RJ, Naj AC, Moore JH. Genetic heterogeneity: challenges, impacts, and methods through an associative lens. Genet Epidemiol (2022) 46(8):555–71. doi: 10.1002/gepi.22497 35924480 PMC9669229

[44] Simonetto C, Rospleszcz S, Kaiser JC, Furukawa K. Heterogeneity in coronary heart disease risk. Sci Rep (2022) 12(1):10131. doi: 10.1038/s41598-022-14013-3 35710917 PMC9203574

[45] Watkin DM, Lawry EY, Mann GV, Halperin M. A study of serum beta lipoprotein and total cholesterol variability and its relation to age and serum level in adult human subjects. J Clin Invest (1954) 33(6):874–83. doi: 10.1172/JCI102960 13163180 PMC438523

[46] Castelli WP, Doyle JT, Gordon T, Hames CG, Hjortland MC, Hulley SB, HDL cholesterol and other lipids in coronary heart disease. The cooperative lipoprotein phenotyping study. Circulation (1977) 55(5):767–72. doi: 10.1161/01.cir.55.5.767 191215

[47] Castelli WP, Cooper GR, Doyle JT, Garcia-Palmieri M, Gordon T, Hames C, Distribution of triglyceride and total, LDL and HDL cholesterol in several populations: a cooperative lipoprotein phenotyping study. J Chronic Dis (1977) 30(3):147–69. doi: 10.1016/0021-9681(77)90082-0 191465

[48] Ridker PM. Evaluating novel cardiovascular risk factors: can we better predict heart attacks? Ann Intern Med (1999) 130(11):933–7. doi: 10.7326/0003-4819-130-11-199906010-00018 10375342

[49] Crea F, Libby P. Acute coronary syndromes: the way forward from mechanisms to precision treatment. Circulation (2017) 136(12):1155–66. doi: 10.1161/CIRCULATIONAHA.117.029870 28923905 PMC5679086

[50] Dai X, Wiernek S, Evans JP, Runge MS. Genetics of coronary artery disease and myocardial infarction. World J Cardiol (2016) 8(1):1–23. doi: 10.4330/wjc.v8.i1.1 26839654 PMC4728103

[51] Kaski JC, Crea F, Gersh BJ, Camici PG. Reappraisal of ischemic heart disease. Circulation (2018) 138(14):1463–80. doi: 10.1161/CIRCULATIONAHA.118.031373 30354347

[52] Maron BJ, Maron MS, Maron BA, Loscalzo J. Moving beyond the sarcomere to explain heterogeneity in hypertrophic cardiomyopathy: JACC review topic of the week. J Am Coll Cardiol (2019) 73(15):1978–86. doi: 10.1016/j.jacc.2019.01.061 31000001 PMC6550351

[53] Peters AE, Tromp J, Shah SJ, Lam CSP, Lewis GD, Borlaug BA, Phenomapping in heart failure with preserved ejection fraction: insights, limitations, and future directions. Cardiovasc Res (2023) 118(18):3403–15. doi: 10.1093/cvr/cvac179 36448685 PMC10144733

[54] Simmonds SJ, Cuijpers I, Heymans S, Jones EAV. Cellular and molecular differences between HFpEF and HFrEF: a step ahead in an improved pathological understanding. Cells (2020) 9(1):242. doi: 10.3390/cells9010242 31963679 PMC7016826

[55] Liu Y, Chen Y, Wei B, Li H, Peng Y, Luo Z. Impacts of ABCG2 loss of function variant (p. Gln141Lys, c.421 C > A, rs2231142) on lipid levels and statin efficiency: a systematic review and meta-analysis. BMC Cardiovasc Disord (2024) 24(1):202. doi: 10.1186/s12872-024-03821-2 38589776 PMC11000409

[56] Ingelman-Sundberg M, Pirmohamed M. Precision medicine in cardiovascular therapeutics: evaluating the role of pharmacogenetic analysis prior to drug treatment. J Intern Med (2024) 295(5):583–98. doi: 10.1111/joim.13772 38343077

[57] Chan MY, Andreotti F, Becker RC. Hypercoagulable states in cardiovascular disease. Circulation (2008) 118(22):2286–97. doi: 10.1161/CIRCULATIONAHA.108.778837 19029477

[58] Fernandez DM, Rahman AH, Fernandez NF, Chudnovskiy A, Amir ED, Amadori L, Single-cell immune landscape of human atherosclerotic plaques. Nat Med (2019) 25(10):1576–88. doi: 10.1038/s41591-019-0590-4 31591603 PMC7318784

[59] Depuydt MAC, Prange KHM, Slenders L, Örd T, Elbersen D, Boltjes A, Microanatomy of the human atherosclerotic plaque by single-cell transcriptomics. Circ Res (2020) 127(11):1437–55. doi: 10.1161/CIRCRESAHA.120.316770 32981416 PMC7641189

[60] Lin JD, Nishi H, Poles J, Niu X, McCauley C, Rahman K, Single-cell analysis of fate-mapped macrophages reveals heterogeneity, including stem-like properties, during atherosclerosis progression and regression. JCI Insight (2019) 4(4):e124574. doi: 10.1172/jci.insight.124574 30830865 PMC6478411

[61] Vallejo J, Cochain C, Zernecke A, Ley K. Heterogeneity of immune cells in human atherosclerosis revealed by scRNA-Seq. Cardiovasc Res (2021) 117(13):2537–43. doi: 10.1093/cvr/cvab260 34343272 PMC8921647

[62] Pan H, Xue C, Auerbach BJ, Fan J, Bashore AC, Cui J, Single-cell genomics reveals a novel cell state during smooth muscle cell phenotypic switching and potential therapeutic targets for atherosclerosis in mouse and human. Circulation (2020) 142(21):2060–75. doi: 10.1161/CIRCULATIONAHA.120.048378 32962412 PMC8104264

[63] Kalluri AS, Vellarikkal SK, Edelman ER, Nguyen L, Subramanian A, Ellinor PT, Single-cell analysis of the normal mouse aorta reveals functionally distinct endothelial cell populations. Circulation (2019) 140(2):147–63. doi: 10.1161/CIRCULATIONAHA.118.038362 31146585 PMC6693656

[64] Winkels H, Ehinger E, Vassallo M, Buscher K, Dinh HQ, Kobiyama K, Atlas of the immune cell repertoire in mouse atherosclerosis defined by single-cell RNA-sequencing and mass cytometry. Circ Res (2018) 122(12):1675–88. doi: 10.1161/CIRCRESAHA.117.312513 29545366 PMC5993603

[65] McQueen LW, Ladak SS, Abbasciano R, George SJ, Suleiman MS, Angelini GD, Next-generation and single-cell sequencing approaches to study atherosclerosis and vascular inflammation pathophysiology: a systematic review. Front Cardiovasc Med (2022) 9:849675. doi: 10.3389/fcvm.2022.849675 35419441 PMC8996078

[66] Sonawane AR, Pucéat M, Jo H. Single-cell OMICs analyses in cardiovascular diseases. Front Cardiovasc Med (2024) 11:1413184. doi: 10.3389/fcvm.2024.1413184 38770014 PMC11102967

[67] Decano JL, Aikawa M. Dynamic macrophages: understanding mechanisms of activation as guide to therapy for atherosclerotic vascular disease. Front Cardiovasc Med (2018) 5:97. doi: 10.3389/fcvm.2018.00097 30123798 PMC6086112

[68] Decano JL, Maiorino E, Matamalas JT, Chelvanambi S, Tiemeijer BM, Yanagihara Y, Cellular heterogeneity of activated primary human macrophages and associated drug-gene networks: from biology to precision therapeutics. Circulation (2023) 148(19):1459–78. doi: 10.1161/CIRCULATIONAHA.123.064794 37850387 PMC10624416

[69] Geyer PE, Holdt LM, Teupser D, Mann M. Revisiting biomarker discovery by plasma proteomics. Mol Syst Biol (2017) 13(9):942. doi: 10.15252/msb.20156297 28951502 PMC5615924

[70] Eldjarn GH, Ferkingstad E, Lund SH, Helgason H, Magnusson OT, Gunnarsdottir K, Large-scale plasma proteomics comparisons through genetics and disease associations. Nature (2023) 622(7982):348–58. doi: 10.1038/s41586-023-06563-x 37794188 PMC10567571

[71] Leopold JA, Maron BA, Loscalzo J. The application of big data to cardiovascular disease: paths to precision medicine. J Clin Invest (2020) 130(1):29–38. doi: 10.1172/JCI129203 31895052 PMC6934200

[72] Mohanta SK, Peng L, Li Y, Lu S, Sun T, Carnevale L, Neuroimmune cardiovascular interfaces control atherosclerosis. Nature (2022) 605(7908):152–9. doi: 10.1038/s41586-022-04673-6 35477759

[73] Caudal A, Snyder MP, Wu JC. Harnessing human genetics and stem cells for precision cardiovascular medicine. Cell Genom (2024) 4(2):100445. doi: 10.1016/j.xgen.2023.100445 38359791 PMC10879032

[74] Atutornu J, Milne R, Costa A, Patch C, Middleton A. Towards equitable and trustworthy genomics research. EBiomedicine (2022) 76:103879. doi: 10.1016/j.ebiom.2022.103879 35158310 PMC8850759

[75] Loscalzo J, Barabási AL, Silverman EK. Network medicine: complex systems in human disease and therapeutics. Cambridge, MA: Harvard University Press (2017).

[76] Lusis AJ, Seldin MM, Allayee H, Bennett BJ, Civelek M, Davis RC, The hybrid mouse diversity panel: a resource for systems genetics analyses of metabolic and cardiovascular traits. J Lipid Res (2016) 57(6):925–42. doi: 10.1194/jlr.R066944 27099397 PMC4878195

[77] Daugherty A, Tall AR, Daemen MJAP, Falk E, Fisher EA, García-Cardeña G, Recommendation on design, execution, and reporting of animal atherosclerosis studies: a scientific statement from the American Heart Association. Arterioscler Thromb Vasc Biol (2017) 37(9):e131–57. doi: 10.1161/ATV.0000000000000062 28729366

[78] Thomas D, Wu JC. Integrative approaches in cardiac tissue engineering: bridging cellular complexity to create accurate physiological models. iScience (2025) 28(8):113003. doi: 10.1016/j.isci.2025.113003 40697825 PMC12281012

[79] Juguilon C, Khosravi R, Radisic M, Wu JC. *In vitro* modeling of interorgan crosstalk: multi-organ-on-a-chip for studying cardiovascular-kidney-metabolic syndrome. Circ Res (2025) 136(11):1476–93. doi: 10.1161/CIRCRESAHA.125.325497 40403116 PMC12180411

[80] Yildirim Z, Swanson K, Wu X, Zou J, Wu J. Next-gen therapeutics: pioneering drug discovery with iPSCs, genomics, AI, and clinical trials in a dish. Annu Rev Pharmacol Toxicol (2025) 65(1):71–90. doi: 10.1146/annurev-pharmtox-022724-095035 39284102 PMC12011342

[81] Wu X, Swanson K, Yildirim Z, Liu W, Liao R, Wu JC. Clinical trials in-a-dish for cardiovascular medicine. Eur Heart J (2024) 45(40):4275–90. doi: 10.1093/eurheartj/ehae519 39270727 PMC11491156

[82] Brown DG, Wobst HJ, Kapoor A, Kenna LA, Southall N. Clinical development times for innovative drugs. Nat Rev Drug Discov (2022) 21(11):793–4. doi: 10.1038/d41573-021-00190-9 34759309 PMC9869766

[83] Mohs RC, Greig NH. Drug discovery and development: role of basic biological research. Alzheimers Dement (2017) 3(4):651–7. doi: 10.1016/j.trci.2017.10.005 PMC5725284 29255791

[84] Sasso JM, Ambrose BJB, Tenchov R, Datta RS, Basel MT, DeLong RK, The progress and promise of RNA medicine─an arsenal of targeted treatments. J Med Chem (2022) 65(10):6975–7015. doi: 10.1021/acs.jmedchem.2c00024 35533054 PMC9115888

[85] Hinkson IV, Madej B, Stahlberg EA. Accelerating therapeutics for opportunities in medicine: a paradigm shift in drug discovery. Front Pharmacol (2020) 11:770. doi: 10.3389/fphar.2020.00770 32694991 PMC7339658

[86] Pilkington EH, Suys EJA, Trevaskis NL, Wheatley AK, Zukancic D, Algarni A, From influenza to COVID-19: lipid nanoparticle mRNA vaccines at the frontiers of infectious diseases. Acta Biomater (2021) 131:16–40. doi: 10.1016/j.actbio.2021.06.023 34153512 PMC8272596

[87] Kalinke U, Barouch DH, Rizzi R, Lagkadinou E, Türeci Ö, Pather S, Clinical development and approval of COVID-19 vaccines. Expert Rev Vaccines (2022) 21(5):609–19. doi: 10.1080/14760584.2022.2042257 35157542 PMC8935460

[88] Dowden H, Munro J. Trends in clinical success rates and therapeutic focus. Nat Rev Drug Discov (2019) 18(7):495–6. doi: 10.1038/d41573-019-00074-z 31267067

[89] Takebe T, Imai R, Ono S. The current status of drug discovery and development as originated in United States academia: the influence of industrial and academic collaboration on drug discovery and development. Clin Transl Sci (2018) 11(6):597–606. doi: 10.1111/cts.12577 29940695 PMC6226120

[90] Firoozbakht F, Elkjaer ML, Handy DE, Wang RS, Chervontseva Z, Rarey M, Exploring common mechanisms of adverse drug reactions and disease phenotypes through network-based analysis. Cell Rep Methods (2025) 5(2):100990. doi: 10.1016/j.crmeth.2025.100990 39954672 PMC11955268

[91] Harrison RK. Phase II and phase III failures: 2013–2015. Nat Rev Drug Discov (2016) 15(12):817–8. doi: 10.1038/nrd.2016.184 27811931

[92] Ferdinandy P, Baczkó I, Bencsik P, Giricz Z, Görbe A, Pacher P, Definition of hidden drug cardiotoxicity: paradigm change in cardiac safety testing and its clinical implications. Eur Heart J (2019) 40(22):1771–7. doi: 10.1093/eurheartj/ehy365 29982507 PMC6554653

[93] Moffat JG, Vincent F, Lee JA, Eder J, Prunotto M. Opportunities and challenges in phenotypic drug discovery: an industry perspective. Nat Rev Drug Discov (2017) 16(8):531–43. doi: 10.1038/nrd.2017.111 28685762

[94] Iwata H, Goettsch C, Sharma A, Ricchiuto P, Goh WWB, Halu A, PARP9 and PARP14 cross-regulate macrophage activation via STAT1 ADP-ribosylation. Nat Commun (2016) 7:12849. doi: 10.1038/ncomms12849 27796300 PMC5095532

[95] Nakano T, Katsuki S, Chen M, Decano JL, Halu A, Lee LH, Uremic toxin indoxyl sulfate promotes proinflammatory macrophage activation via the interplay of OATP2B1 and Dll4-notch signaling. Circulation (2019) 139(1):78–96. doi: 10.1161/CIRCULATIONAHA.118.034588 30586693 PMC6311723

[96] Paci P, Fiscon G, Conte F, Wang RS, Handy DE, Farina L, Comprehensive network medicine-based drug repositioning via integration of therapeutic efficacy and side effects. NPJ Syst Biol Appl (2022) 8(1):12. doi: 10.1038/s41540-022-00221-0 35443763 PMC9021283

[97] Gardiner LJ, Carrieri AP, Wilshaw J, Checkley S, Pyzer-Knapp EO, Krishna R. Using human *in vitro* transcriptome analysis to build trustworthy machine learning models for prediction of animal drug toxicity. Sci Rep (2020) 10(1):9522. doi: 10.1038/s41598-020-66481-0 32533004 PMC7293302

[98] Fiscon G, Conte F, Farina L, Paci P. A comparison of network-based methods for drug repurposing along with an application to human complex diseases. Int J Mol Sci (2022) 23(7):3703. doi: 10.3390/ijms23073703 35409062 PMC8999012

[99] Finan C, Gaulton A, Kruger FA, Lumbers RT, Shah T, Engmann J, The druggable genome and support for target identification and validation in drug development. Sci Transl Med (2017) 9(383):eaag1166. doi: 10.1126/scitranslmed.aag1166 PMC6321762 28356508

[100] Hopkins AL, Groom CR. The druggable genome. Nat Rev Drug Discov (2002) 1(9):727–30. doi: 10.1038/nrd892 12209152

[101] Hopkins AL, Keserü GM, Leeson PD, Rees DC, Reynolds CH. The role of ligand efficiency metrics in drug discovery. Nat Rev Drug Discov (2014) 13(2):105–21. doi: 10.1038/nrd4163 24481311

[102] Tanaka T, Asano T, Okui T, Kuraoka S, Singh SA, Aikawa M, Computational screening strategy for drug repurposing identified niclosamide as inhibitor of vascular calcification. Front Cardiovasc Med (2021) 8:826529. doi: 10.3389/fcvm.2021.826529 35127876 PMC8811128

[103] Asano T, Chelvanambi S, Decano JL, Whelan MC, Aikawa E, Aikawa M. In silico drug screening approach using L1000-based connectivity map and its application to COVID-19. Front Cardiovasc Med (2022) 9:842641. doi: 10.3389/fcvm.2022.842641 35402570 PMC8989014

[104] Crooke ST, Witztum JL, Bennett CF, Baker BF. RNA-targeted therapeutics. Cell Metab (2018) 27(4):714–39. doi: 10.1016/j.cmet.2018.03.004 29617640

[105] Kim YK. RNA therapy: rich history, various applications and unlimited future prospects. Exp Mol Med (2022) 54(4):455–65. doi: 10.1038/s12276-022-00757-5 35440755 PMC9016686

[106] Krychtiuk KA, Rader DJ, Granger CB. RNA-targeted therapeutics in cardiovascular disease: the time is now. Eur Heart J Cardiovasc Pharmacother (2022) 9(1):94–9. doi: 10.1093/ehjcvp/pvac052 36138490

[107] Agache I, Akdis CA. Precision medicine and phenotypes, endotypes, genotypes, regiotypes, and theratypes of allergic diseases. J Clin Invest (2019) 129(4):1493–503. doi: 10.1172/JCI124611 30855278 PMC6436902

[108] Kitano H Systems biology: a brief overview. Science (2002) 295(5560):1662–4. doi: 10.1126/science.1069492 11872829

[109] Loscalzo J, Barabasi AL. Systems biology and the future of medicine. Wiley Interdiscip Rev Syst Biol Med (2011) 3(6):619–27. doi: 10.1002/wsbm.144 21928407 PMC3188693

[110] Schork NJ. Personalized medicine: time for one-person trials. Nature (2015) 520(7549):609–11. doi: 10.1038/520609a 25925459

[111] Lee LY, Pandey AK, Maron BA, Loscalzo J. Network medicine in cardiovascular Research. Cardiovasc Res (2021) 117(10):2186–202. doi: 10.1093/cvr/cvaa321 33165538 PMC8404464

[112] Sonawane AR, Weiss ST, Glass K, Sharma A. Network medicine in the age of biomedical big data. Front Genet (2019) 10:294. doi: 10.3389/fgene.2019.00294 31031797 PMC6470635

[113] Ahn AC, Tewari M, Poon CS, Phillips RS. The limits of reductionism in medicine: could systems biology offer an alternative? PloS Med (2006) 3(6):e208. doi: 10.1371/journal.pmed.0030208 16681415 PMC1459480

[114] Ahn AC, Tewari M, Poon CS, Phillips RS. The clinical applications of a systems approach. PloS Med (2006) 3(7):e209. doi: 10.1371/journal.pmed.0030209 16683861 PMC1459481

[115] Wolkenhauer O, Auffray C, Jaster R, Steinhoff G, Dammann O. The road from systems biology to systems medicine. Pediatr Res (2013) 73(4 Pt 2):502–7. doi: 10.1038/pr.2013.4 23314297

[116] Clermont G, Auffray C, Moreau Y, Rocke DM, Dalevi D, Dubhashi D, Bridging the gap between systems biology and medicine. Genome Med (2009) 1(9):88. doi: 10.1186/gm88 19754960 PMC2768995

[117] Barabási AL, Gulbahce N, Loscalzo J. Network medicine: a network-based approach to human disease. Nat Rev Genet (2011) 12(1):56–68. doi: 10.1038/nrg2918 21164525 PMC3140052

[118] Lusis AJ, Weiss JN. Cardiovascular networks: systems-based approaches to cardiovascular disease. Circulation (2010) 121(1):157–70. doi: 10.1161/CIRCULATIONAHA.108.847699 20048233 PMC2836123

[119] MacLellan WR, Wang Y, Lusis AJ. Systems-based approaches to cardiovascular disease. Nat Rev Cardiol (2012) 9(3):172–84. doi: 10.1038/nrcardio.2011.208 22231714 PMC3823242

[120] Sarajlić A, Pržulj N. Survey of network-based approaches to research of cardiovascular diseases. BioMed Res Int (2014) 2014:527029. doi: 10.1155/2014/527029 24772427 PMC3977459

[121] Zhao Y, Chen J, Freudenberg JM, Meng Q, Rajpal DK, Yang X. Network-based identification and prioritization of key regulators of coronary artery disease loci. Arterioscler Thromb Vasc Biol (2016) 36(5):928–41. doi: 10.1161/ATVBAHA.115.306725 26966275 PMC5576868

[122] King JY, Ferrara R, Tabibiazar R, Spin JM, Chen MM, Kuchinsky A, Pathway analysis of coronary atherosclerosis. Physiol Genomics (2005) 23(1):103–18. doi: 10.1152/physiolgenomics.00101.2005 15942018

[123] Ashley EA, Ferrara R, King JY, Vailaya A, Kuchinsky A, He X, Network analysis of human in-stent restenosis. Circulation (2006) 114(24):2644–54. doi: 10.1161/CIRCULATIONAHA.106.637025 17145989

[124] Schlesinger J, Schueler M, Grunert M, Fischer JJ, Zhang Q, Krueger T, The cardiac transcription network modulated by Gata4, Mef2a, Nkx2.5, Srf, histone modifications, and microRNAs. PloS Genet (2011) 7(2):e1001313. doi: 10.1371/journal.pgen.1001313 21379568 PMC3040678

[125] Skogsberg J, Lundström J, Kovacs A, Nilsson R, Noori P, Maleki S, Transcriptional profiling uncovers a network of cholesterol-responsive atherosclerosis target genes. PloS Genet (2008) 4(3):e1000036. doi: 10.1371/journal.pgen.1000036 18369455 PMC2265530

[126] Jin G, Zhou X, Wang H, Zhao H, Cui K, Zhang XS, The knowledge-integrated network biomarkers discovery for major adverse cardiac events. J Proteome Res (2008) 7(9):4013–21. doi: 10.1021/pr8002886 18665624 PMC2854538

[127] Camargo A, Azuaje F. Linking gene expression and functional network data in human heart failure. PloS One (2007) 2(12):e1347. doi: 10.1371/journal.pone.0001347 18094754 PMC2147076

[128] Camargo A, Azuaje F. Identification of dilated cardiomyopathy signature genes through gene expression and network data integration. Genomics (2008) 92(6):404–13. doi: 10.1016/j.ygeno.2008.05.007 18595652

[129] Schlotter F, Halu A, Goto S, Blaser MC, Body SC, Lee LH, Spatiotemporal multi-omics mapping generates a molecular atlas of the aortic valve and reveals networks driving disease. Circulation (2018) 138(4):377–93. doi: 10.1161/CIRCULATIONAHA.117.032291 29588317 PMC6160370

[130] Decano JL, Iwamoto Y, Goto S, Lee JY, Matamalas JT, Halu A, A disease-driver population within interstitial cells of human calcific aortic valves identified via single-cell and proteomic profiling. Cell Rep (2022) 39(2):110685. doi: 10.1016/j.celrep.2022.110685 35417712

[131] Blaser MC, Buffolo F, Halu A, Turner ME, Schlotter F, Higashi H, Multiomics of tissue extracellular vesicles identifies unique modulators of atherosclerosis and calcific aortic valve stenosis. Circulation (2023) 148(8):661–78. doi: 10.1161/CIRCULATIONAHA.122.063402 37427430 PMC10527599

[132] Karlstädt A, Fliegner D, Kararigas G, Ruderisch HS, Regitz-Zagrosek V, Holzhütter HG. CardioNet: a human metabolic network suited for the study of cardiomyocyte metabolism. BMC Syst Biol (2012) 6:114. doi: 10.1186/1752-0509-6-114 22929619 PMC3568067

[133] Ntalla I, Weng LC, Cartwright JH, Hall AW, Sveinbjornsson G, Tucker NR, Multi-ancestry GWAS of the electrocardiographic PR interval identifies 202 loci underlying cardiac conduction. Nat Commun (2020) 11(1):2542. doi: 10.1038/s41467-020-15706-x 32439900 PMC7242331

[134] van Setten J, Verweij N, Mbarek H, Niemeijer MN, Trompet S, Arking DE, Genome-wide association meta-analysis of 30,000 samples identifies seven novel loci for quantitative ECG traits. Eur J Hum Genet (2019) 27(6):952–62. doi: 10.1038/s41431-018-0295-z 30679814 PMC6777533

[135] Roselli C, Rienstra M, Ellinor PT. Genetics of atrial fibrillation in 2020: GWAS, genome sequencing, polygenic risk, and beyond. Circ Res (2020) 127(1):21–33. doi: 10.1161/CIRCRESAHA.120.316575 32716721 PMC7388073

[136] Assum I, Krause J, Scheinhardt MO, Müller C, Hammer E, Börschel CS, Tissue-specific multi-omics analysis of atrial fibrillation. Nat Commun (2022) 13(1):441. doi: 10.1038/s41467-022-27953-1 35064145 PMC8782899

[137] Diez D, Wheelock AM, Goto S, Haeggström JZ, Paulsson-Berne G, Hansson GK, The use of network analyses for elucidating mechanisms in cardiovascular disease. Mol Biosyst (2010) 6(2):289–304. doi: 10.1039/b912078e 20094647

[138] Wheelock CE, Wheelock AM, Kawashima S, Diez D, Kanehisa M, van Erk M, Systems biology approaches and pathway tools for investigating cardiovascular disease. Mol Biosyst (2009) 5(6):588–602. doi: 10.1039/b902356a 19462016

[139] He D, Liu ZP, Chen L. Identification of dysfunctional modules and disease genes in congenital heart disease by a network-based approach. BMC Genomics (2011) 12:592. doi: 10.1186/1471-2164-12-592 22136190 PMC3256240

[140] Dewey FE, Perez MV, Wheeler MT, Watt C, Spin J, Langfelder P, Gene coexpression network topology of cardiac development, hypertrophy, and failure. Circ Cardiovasc Genet (2011) 4(1):26–35. doi: 10.1161/CIRCGENETICS.110.941757 21127201 PMC3324316

[141] Zhang L, Li X, Tai J, Li W, Chen L. Predicting candidate genes based on combined network topological features: a case study in coronary artery disease. PloS One (2012) 7(6):e39542. doi: 10.1371/journal.pone.0039542 22761820 PMC3382204

[142] Sarajlić A, Janjić V, Stojković N, Radak D, Pržulj N. Network topology reveals key cardiovascular disease genes. PloS One (2013) 8(8):e71537. doi: 10.1371/journal.pone.0071537 23977067 PMC3744556

[143] Katsuki S, K Jha P, Lupieri A, Nakano T, Passos LSA, Rogers MA, Proprotein convertase subtilisin/kexin 9 (PCSK9) promotes macrophage activation via LDL receptor-independent mechanisms. Circ Res (2022) 131(11):873–89. doi: 10.1161/CIRCRESAHA.121.320056 36263780 PMC9973449

[144] Decano JL, Singh SA, Gasparotto Bueno C, Ho Lee L, Halu A, Chelvanambi S, Systems approach to discovery of therapeutic targets for vein graft disease: PPARα pivotally regulates metabolism, activation, and heterogeneity of macrophages and lesion development. Circulation (2021) 143(25):2454–70. doi: 10.1161/CIRCULATIONAHA.119.043724 33821665 PMC8212880

[145] Okui T, Iwashita M, Rogers MA, Halu A, Atkins SK, Kuraoka S, CROT (carnitine O-octanoyltransferase) is a novel contributing factor in vascular calcification via promoting fatty acid metabolism and mitochondrial dysfunction. Arterioscler Thromb Vasc Biol (2021) 41(2):755–68. doi: 10.1161/ATVBAHA.120.315007 33356393 PMC8105275

[146] Passos LSA, Becker-Greene D, Braulio R, Le TD, Gelape CL, de Almeida LFR, Proinflammatory matrix metalloproteinase-1 associates with mitral valve leaflet disruption following percutaneous mitral valvuloplasty. Front Cardiovasc Med (2021) 8:804111. doi: 10.3389/fcvm.2021.804111 35127864 PMC8811173

[147] Clift CL, Blaser MC, Gerrits W, Turner ME, Sonawane A, Pham T, Intracellular proteomics and extracellular vesiculomics as a metric of disease recapitulation in 3D-bioprinted aortic valve arrays. Sci Adv (2024) 10(9):eadj9793. doi: 10.1126/sciadv.adj9793 PMC10901368 38416823

[148] Morgan S, Lee LH, Halu A, Nicolau JS, Higashi H, Ha AH, Identifying novel mechanisms of abdominal aortic aneurysm via unbiased proteomics and systems biology. Front Cardiovasc Med (2022) 9:889994. doi: 10.3389/fcvm.2022.889994 35990960 PMC9382335

[149] Huan T, Esko T, Peters MJ, Pilling LC, Schramm K, Schurmann C, A meta-analysis of gene expression signatures of blood pressure and hypertension. PloS Genet (2015) 11(3):e1005035. doi: 10.1371/journal.pgen.1005035 25785607 PMC4365001

[150] Huan T, Meng Q, Saleh MA, Norlander AE, Joehanes R, Zhu J, Integrative network analysis reveals molecular mechanisms of blood pressure regulation. Mol Syst Biol (2015) 11(1):799. doi: 10.15252/msb.20145399 25882670 PMC4422556

[151] Frades I, Readhead B, Amadori L, Koplev S, Talukdar HA, Crane HM, Systems pharmacology identifies an arterial wall regulatory gene network mediating coronary artery disease side effects of antiretroviral therapy. Circ Genom Precis Med (2019) 12(6):e002390. doi: 10.1161/CIRCGEN.118.002390 31059280 PMC6601350

[152] Halu A, De Domenico M, Arenas A, Sharma A. The multiplex network of human diseases. NPJ Syst Biol Appl (2019) 5:15. doi: 10.1038/s41540-019-0092-5 31044086 PMC6478736

[153] Liu X, Maiorino E, Halu A, Glass K, Prasad RB, Loscalzo J, Robustness and lethality in multilayer biological molecular networks. Nat Commun (2020) 11(1):6043. doi: 10.1038/s41467-020-19841-3 33247151 PMC7699651

[154] Maiorino E, Loscalzo J. Phenomics and robust multiomics data for cardiovascular disease subtyping. Arterioscler Thromb Vasc Biol (2023) 43(7):1111–23. doi: 10.1161/ATVBAHA.122.318892 37226730 PMC10330619

[155] Wang RS, Maron BA, Loscalzo J. Multiomics network medicine approaches to precision medicine and therapeutics in cardiovascular diseases. Arterioscler Thromb Vasc Biol (2023) 43(4):493–503. doi: 10.1161/ATVBAHA.122.318731 36794589 PMC10038904

[156] Loscalzo J Molecular interaction networks and drug development: novel approach to drug target identification and drug repositioning. FASEB J (2023) 37(1):e22660. doi: 10.1096/fj.202201683R 36468661 PMC10107166

[157] Blaser MC, Kraler S, Lüscher TF, Aikawa E. Multi-omics approaches to define calcific aortic valve disease pathogenesis. Circ Res (2021) 128(9):1371–97. doi: 10.1161/CIRCRESAHA.120.317979 33914608 PMC8095729

[158] Wang Z, Clark NR, Ma’ayan A. Drug-induced adverse events prediction with the Lincs L1000 data. Bioinformatics (2016) 32(15):2338–45. doi: 10.1093/bioinformatics/btw168 27153606 PMC4965635

[159] Ghiassian SD, Menche J, Chasman DI, Giulianini F, Wang R, Ricchiuto P, Endophenotype Network models: common core of complex diseases. Sci Rep (2016) 6:27414. doi: 10.1038/srep27414 27278246 PMC4899691

[160] Menche J, Sharma A, Kitsak M, Ghiassian SD, Vidal M, Loscalzo J, Disease networks. Uncovering disease-disease relationships through the incomplete interactome. Science (2015) 347(6224):1257601. doi: 10.1126/science.1257601 25700523 PMC4435741

[161] Halu A, Wang JG, Iwata H, Mojcher A, Abib AL, Singh SA, Context-enriched interactome powered by proteomics helps the identification of novel regulators of macrophage activation. eLife (2018) 7:e37059. doi: 10.7554/eLife.37059 30303482 PMC6179386

[162] Yang X Multitissue multiomics systems biology to dissect complex diseases. Trends Mol Med (2020) 26(8):718–28. doi: 10.1016/j.molmed.2020.04.006 32439301 PMC7395877

[163] Morgan NV. Editorial: case reports in cardiovascular genetics and systems medicine: 2022. Front Cardiovasc Med (2023) 10:1282147. doi: 10.3389/fcvm.2023.1282147 37767370 PMC10520268

[164] Jordan E, Peterson L, Ai T, Asatryan B, Bronicki L, Brown E, Evidence-based assessment of genes in dilated cardiomyopathy. Circulation (2021) 144(1):7–19. doi: 10.1161/CIRCULATIONAHA.120.053033 33947203 PMC8247549

[165] Stuart T, Satija R. Integrative single-cell analysis. Nat Rev Genet (2019) 20(5):257–72. doi: 10.1038/s41576-019-0093-7 30696980

[166] Crosetto N, Bienko M, van Oudenaarden A. Spatially resolved transcriptomics and beyond. Nat Rev Genet (2015) 16(1):57–66. doi: 10.1038/nrg3832 25446315

[167] Joshi A, Rienks M, Theofilatos K, Mayr M. Systems biology in cardiovascular disease: a multiomics approach. Nat Rev Cardiol (2021) 18(5):313–30. doi: 10.1038/s41569-020-00477-1 33340009

[168] Skelly DA, Squiers GT, McLellan MA, Bolisetty MT, Robson P, Rosenthal NA, Single-cell transcriptional profiling reveals cellular diversity and intercommunication in the mouse heart. Cell Rep (2018) 22(3):600–10. doi: 10.1016/j.celrep.2017.12.072 29346760

[169] Amadori L, Calcagno C, Fernandez DM, Koplev S, Fernandez N, Kaur R, Systems immunology-based drug repurposing framework to target inflammation in atherosclerosis. Nat Cardiovasc Res (2023) 2(6):550–71. doi: 10.1038/s44161-023-00278-y 37771373 PMC10538622

[170] Teschendorff AE, Feinberg AP. Statistical mechanics meets single-cell biology. Nat Rev Genet (2021) 22(7):459–76. doi: 10.1038/s41576-021-00341-z 33875884 PMC10152720

[171] Hamilton WL, Ying R, Leskovec J. Representation learning on graphs: methods and applications. IEEE Data Eng Bull (2017) 40(3):52–74. Available at: http://sites.computer.org/debull/A17sept/p52.pdf

[172] Zitnik M, Nguyen F, Wang B, Leskovec J, Goldenberg A, Hoffman MM. Machine learning for integrating data in biology and medicine: principles, practice, and opportunities. Inf Fusion (2019) 50:71–91. doi: 10.1016/j.inffus.2018.09.012 30467459 PMC6242341

[173] Huang K, Fu T, Gao W, Zhao Y, Roohani Y, Leskovec J, Artificial intelligence foundation for therapeutic science. Nat Chem Biol (2022) 18(10):1033–6. doi: 10.1038/s41589-022-01131-2 36131149 PMC9529840

[174] Ballantyne CM. Newer risk markers and surrogate endpoints in atherosclerosis management. Clin Cardiol (2001) 24(S3):13–7. doi: 10.1002/clc.4960241504 PMC6655004 11501598

[175] Stanley BA, Gundry RL, Cotter RJ, Van Eyk JE. Heart disease, clinical proteomics and mass spectrometry. Dis Markers (2004) 20(3):167–78. doi: 10.1155/2004/965261 15502250 PMC3839266

[176] Pape ME, Bisgaier CL. Discovering HDL-elevating drugs: are there simplistic approaches to a polygenic disorder? IDrugs (1998) 1(4):442–51 18465577

[177] Singh SA, Aikawa E, Aikawa M. Current trends and future perspectives of state-of-the-art proteomics technologies applied to cardiovascular disease research. Circ J (2016) 80(8):1674–83. doi: 10.1253/circj.CJ-16-0499 27430298

[178] McGarrah RW, Crown SB, Zhang GF, Shah SH, Newgard CB. Cardiovascular metabolomics. Circ Res (2018) 122(9):1238–58. doi: 10.1161/CIRCRESAHA.117.311002 29700070 PMC6029726

[179] Barallobre-Barreiro J, Chung YL, Mayr M. Proteomics and metabolomics for mechanistic insights and biomarker discovery in cardiovascular disease. Rev Esp Cardiol (Engl Ed) (2013) 66(8):657–61. doi: 10.1016/j.rec.2013.04.009 24776335

[180] Wirka RC, Pjanic M, Quertermous T. Advances in transcriptomics: investigating cardiovascular disease at unprecedented resolution. Circ Res (2018) 122(9):1200–20. doi: 10.1161/CIRCRESAHA.117.310910 29700068 PMC7274217

[181] Leopold JA, Loscalzo J. Emerging role of precision medicine in cardiovascular disease. Circ Res (2018) 122(9):1302–15. doi: 10.1161/CIRCRESAHA.117.310782 29700074 PMC6021027

[182] Vidal M, Chan DW, Gerstein M, Mann M, Omenn GS, Tagle D, The human proteome - a scientific opportunity for transforming diagnostics, therapeutics, and healthcare. Clin Proteomics (2012) 9(1):6. doi: 10.1186/1559-0275-9-6 22583803 PMC3388576

[183] Herrington DM, Mao C, Parker SJ, Fu Z, Yu G, Chen L, Proteomic architecture of human coronary and aortic atherosclerosis. Circulation (2018) 137(25):2741–56. doi: 10.1161/CIRCULATIONAHA.118.034365 29915101 PMC6011234

[184] Vogel C, Marcotte EM. Insights into the regulation of protein abundance from proteomic and transcriptomic analyses. Nat Rev Genet (2012) 13(4):227–32. doi: 10.1038/nrg3185 22411467 PMC3654667

[185] Kustatscher G, Grabowski P, Rappsilber J. Pervasive coexpression of spatially proximal genes is buffered at the protein level. Mol Syst Biol (2017) 13(8):937. doi: 10.15252/msb.20177548 28835372 PMC5572396

[186] Slavov N Unlocking the potential of single-cell omics. J Proteome Res (2025) 24(4):1481. doi: 10.1021/acs.jproteome.5c00197 40181710 PMC12054357

[187] Kelly RT. Single-cell proteomics: progress and prospects. Mol Cell Proteomics (2020) 19(11):1739–48. doi: 10.1074/mcp.R120.002234 32847821 PMC7664119

[188] Reyes-Soffer G, Millar JS, Ngai C, Jumes P, Coromilas E, Asztalos B, Cholesteryl ester transfer protein inhibition with anacetrapib decreases fractional clearance rates of high-density lipoprotein apolipoprotein A-I and plasma cholesteryl ester transfer protein. Arterioscler Thromb Vasc Biol (2016) 36(5):994–1002. doi: 10.1161/ATVBAHA.115.306680 26966279 PMC4911016

[189] Lassman ME, McAvoy T, Lee AYH, Chappell D, Wong O, Zhou H, Practical immunoaffinity-enrichment LC-MS for measuring protein kinetics of low-abundance proteins. Clin Chem (2014) 60(9):1217–24. doi: 10.1373/clinchem.2014.222455 24751376 PMC4955776

[190] Millar JS, Reyes-Soffer G, Jumes P, Dunbar RL, deGoma EM, Baer AL, Anacetrapib lowers LDL by increasing ApoB clearance in mildly hypercholesterolemic subjects. J Clin Invest (2015) 125(6):2510–22. doi: 10.1172/JCI80025 25961461 PMC4497759

[191] Singh SA, Andraski AB, Pieper B, Goh W, Mendivil CO, Sacks FM, Multiple apolipoprotein kinetics measured in human HDL by high-resolution/accurate mass parallel reaction monitoring. J Lipid Res (2016) 57(4):714–28. doi: 10.1194/jlr.D061432 26862155 PMC4808760

[192] Singh SA, Andraski AB, Higashi H, Lee LH, Ramsaroop A, Sacks FM, Metabolism of PLTP, CETP, and LCAT on multiple HDL sizes using the Orbitrap Fusion Lumos. JCI Insight (2021) 6(3):e143526. doi: 10.1172/jci.insight.143526 33351780 PMC7934878

[193] Smith LM, Agar JN, Chamot-Rooke J, Danis PO, Ge Y, Loo JA, The Human Proteoform Project: defining the human proteome. Sci Adv (2021) 7(46):eabk0734. doi: 10.1126/sciadv.abk0734 PMC8589312 34767442

[194] Moore KJ, Koplev S, Fisher EA, Tabas I, Björkegren JLM, Doran AC, Macrophage trafficking, inflammatory resolution, and genomics in atherosclerosis: JACC macrophage in CVD series (Part 2). J Am Coll Cardiol (2018) 72(18):2181–97. doi: 10.1016/j.jacc.2018.08.2147 30360827 PMC6522246

[195] Kasikara C, Doran AC, Cai B, Tabas I. The role of non-resolving inflammation in atherosclerosis. J Clin Invest (2018) 128(7):2713–23. doi: 10.1172/JCI97950 30108191 PMC6025992

[196] Galindo CL, Khan S, Zhang X, Yeh YS, Liu Z, Razani B. Lipid-laden foam cells in the pathology of atherosclerosis: shedding light on new therapeutic targets. Expert Opin Ther Targets (2023) 27(12):1231–45. doi: 10.1080/14728222.2023.2288272 38009300 PMC10843715

[197] Mantovani A, Sozzani S, Locati M, Allavena P, Sica A. Macrophage polarization: tumor-associated macrophages as a paradigm for polarized M2 mononuclear phagocytes. Trends Immunol (2002) 23(11):549–55. doi: 10.1016/s1471-4906(02)02302-5 12401408

[198] Gordon S, Martinez FO. Alternative activation of macrophages: mechanism and functions. Immunity (2010) 32(5):593–604. doi: 10.1016/j.immuni.2010.05.007 20510870

[199] Nahrendorf M, Swirski FK. Abandoning M1/M2 for a network model of macrophage function. Circ Res (2016) 119(3):414–17. doi: 10.1161/CIRCRESAHA.116.309194 27458196 PMC4965179

[200] Martinez FO, Gordon S. The M1 and M2 paradigm of macrophage activation: time for reassessment. F1000Prime Rep (2014) 6:13. doi: 10.12703/P6-13 24669294 PMC3944738

[201] Bronte V, Brandau S, Chen SH, Colombo MP, Frey AB, Greten TF, Recommendations for myeloid-derived suppressor cell nomenclature and characterization standards. Nat Commun (2016) 7:12150. doi: 10.1038/ncomms12150 27381735 PMC4935811

[202] Kumar Jha P, Aikawa M, Aikawa E. Macrophage heterogeneity and efferocytosis: beyond the M1/M2 dichotomy. Circ Res (2024) 134(2):186–8. doi: 10.1161/CIRCRESAHA.123.324011 38236949 PMC10798221

[203] Revelo XS, Parthiban P, Chen C, Barrow F, Fredrickson G, Wang H, Cardiac resident macrophages prevent fibrosis and stimulate angiogenesis. Circ Res (2021) 129(12):1086–101. doi: 10.1161/CIRCRESAHA.121.319737 34645281 PMC8638822

[204] Jia G, Preussner J, Chen X, Guenther S, Yuan X, Yekelchyk M, Single cell RNA-seq and ATAC-seq analysis of cardiac progenitor cell transition states and lineage settlement. Nat Commun (2018) 9(1):4877. doi: 10.1038/s41467-018-07307-6 30451828 PMC6242939

[205] Wang L, Yang Y, Ma H, Xie Y, Xu J, Near D, Single-cell dual-omics reveals the transcriptomic and epigenomic diversity of cardiac non-myocytes. Cardiovasc Res (2022) 118(6):1548–63. doi: 10.1093/cvr/cvab134 33839759 PMC9074971

[206] Lilja S, Li X, Smelik M, Lee EJ, Loscalzo J, Marthanda PB, Multi-organ single-cell analysis reveals an on/off switch system with potential for personalized treatment of immunological diseases. Cell Rep Med (2023) 4(3):100956. doi: 10.1016/j.xcrm.2023.100956 36858042 PMC10040389

[207] Leduc A, Khoury L, Cantlon J, Khan S, Slavov N. Massively parallel sample preparation for multiplexed single-cell proteomics using nPOP. Nat Protoc (2024) 19(12):3750–76. doi: 10.1038/s41596-024-01033-8 39117766 PMC11614709

[208] Huffman RG, Leduc A, Wichmann C, Di Gioia M, Borriello F, Specht H, Prioritized mass spectrometry increases the depth, sensitivity and data completeness of single-cell proteomics. Nat Methods (2023) 20(5):714–22. doi: 10.1038/s41592-023-01830-1 37012480 PMC10172113

[209] Hill MC, Kadow ZA, Long H, Morikawa Y, Martin TJ, Birks EJ, Integrated multi-omic characterization of congenital heart disease. Nature (2022) 608(7921):181–91. doi: 10.1038/s41586-022-04989-3 35732239 PMC10405779

[210] Iqbal F, Schlotter F, Becker-Greene D, Lupieri A, Goettsch C, Hutcheson JD, Sortilin enhances fibrosis and calcification in aortic valve disease by inducing interstitial cell heterogeneity. Eur Heart J (2023) 44(10):885–98. doi: 10.1093/eurheartj/ehac818 36660854 PMC9991042

[211] Bartosovic M, Kabbe M, Castelo-Branco G. Single-cell CUT&Tag profiles histone modifications and transcription factors in complex tissues. Nat Biotechnol (2021) 39(7):825–35. doi: 10.1038/s41587-021-00869-9 33846645 PMC7611252

[212] Rodriques SG, Stickels RR, Goeva A, Martin CA, Murray E, Vanderburg CR, Slide-seq: a scalable technology for measuring genome-wide expression at high spatial resolution. Science (2019) 363(6434):1463–7. doi: 10.1126/science.aaw1219 30923225 PMC6927209

[213] Stoeckius M, Hafemeister C, Stephenson W, Houck-Loomis B, Chattopadhyay PK, Swerdlow H, Simultaneous epitope and transcriptome measurement in single cells. Nat Methods (2017) 14(9):865–8. doi: 10.1038/nmeth.4380 28759029 PMC5669064

[214] Tang SS, Guillermier C, Wang M, Poczatek JC, Suzuki N, Loscalzo J, Quantitative imaging of selenoprotein with multi-isotope imaging mass spectrometry (MIMS). Surf Interface Anal (2014) 46(Suppl 1):154–7. doi: 10.1002/sia.5625 26379338 PMC4566158

[215] Cairns JL, Huber J, Lewen A, Jung J, Maurer SJ, Bausbacher T, Mass-guided single-cell MALDI imaging of low-mass metabolites reveals cellular activation markers. Adv Sci (Weinh) (2025) 12(5):e2410506. doi: 10.1002/advs.202410506 39665230 PMC11791930

[216] Forte D, Pellegrino RM, Falvo P, Garcia-Gonzalez P, Alabed HBR, Maltoni F, Parallel single-cell metabolic analysis and extracellular vesicle profiling reveal vulnerabilities with prognostic significance in acute myeloid leukemia. Nat Commun (2024) 15(1):10878. doi: 10.1038/s41467-024-55231-9 39738118 PMC11685939

[217] Rappez L, Stadler M, Triana S, Gathungu RM, Ovchinnikova K, Phapale P, SpaceM reveals metabolic states of single cells. Nat Methods (2021) 18(7):799–805. doi: 10.1038/s41592-021-01198-0 34226721 PMC7611214

[218] Tang F, Barbacioru C, Wang Y, Nordman E, Lee C, Xu N, mRNA-Seq whole-transcriptome analysis of a single cell. Nat Methods (2009) 6(5):377–82. doi: 10.1038/nmeth.1315 19349980

[219] Regev A, Teichmann SA, Lander ES, Amit I, Benoist C, Birney E, The human cell atlas. eLife (2017) 6:e27041. doi: 10.7554/eLife.27041 29206104 PMC5762154

[220] Jovic D, Liang X, Zeng H, Lin L, Xu F, Luo Y. Single-cell RNA sequencing technologies and applications: a brief overview. Clin Transl Med (2022) 12(3):e694. doi: 10.1002/ctm2.694 35352511 PMC8964935

[221] Cusanovich DA, Hill AJ, Aghamirzaie D, Daza RM, Pliner HA, Berletch JB, A single-cell atlas of *in vivo* mammalian chromatin accessibility. Cell (2018) 174(5):1309–24.e18. doi: 10.1016/j.cell.2018.06.052 30078704 PMC6158300

[222] Cao J, Packer JS, Ramani V, Cusanovich DA, Huynh C, Daza R, Comprehensive single-cell transcriptional profiling of a multicellular organism. Science (2017) 357(6352):661–7. doi: 10.1126/science.aam8940 28818938 PMC5894354

[223] Jones RC, Karkanias J, Krasnow MA, Pisco AO, Quake SR, Salzman J, The Tabula Sapiens: a multiple-organ, single-cell transcriptomic atlas of humans. Science (2022) 376(6594):eabl4896. doi: 10.1126/science.abl4896 PMC9812260 35549404

[224] Han X, Zhou Z, Fei L, Sun H, Wang R, Chen Y, Construction of a human cell landscape at single-cell level. Nature (2020) 581(7808):303–9. doi: 10.1038/s41586-020-2157-4 32214235

[225] Kalucka J, de Rooij LPMH, Goveia J, Rohlenova K, Dumas SJ, Meta E, Single-cell transcriptome atlas of murine endothelial cells. Cell (2020) 180(4):764–79.e20. doi: 10.1016/j.cell.2020.01.015 32059779

[226] Schupp JC, Adams TS, Cosme C Jr, Raredon MSB, Yuan Y, Omote N, Integrated single-cell atlas of endothelial cells of the human lung. Circulation (2021) 144(4):286–302. doi: 10.1161/CIRCULATIONAHA.120.052318 34030460 PMC8300155

[227] Travaglini KJ, Nabhan AN, Penland L, Sinha R, Gillich A, Sit RV, A molecular cell atlas of the human lung from single-cell RNA sequencing. Nature (2020) 587(7835):619–25. doi: 10.1038/s41586-020-2922-4 33208946 PMC7704697

[228] Gawel DR, Serra-Musach J, Lilja S, Aagesen J, Arenas A, Asking B, A validated single-cell-based strategy to identify diagnostic and therapeutic targets in complex diseases. Genome Med (2019) 11(1):47. doi: 10.1186/s13073-019-0657-3 31358043 PMC6664760

[229] Lun ATL, McCarthy DJ, Marioni JC. A step-by-step workflow for low-level analysis of single-cell RNA-seq data with bioconductor. F1000Res (2016) 5:2122. doi: 10.12688/f1000research.9501.2 27909575 PMC5112579

[230] Amezquita RA, Lun ATL, Becht E, Carey VJ, Carpp LN, Geistlinger L, Orchestrating single-cell analysis with Bioconductor. Nat Methods (2020) 17(2):137–45. doi: 10.1038/s41592-019-0654-x 31792435 PMC7358058

[231] Xu Y, Tan Y, Zhang X, Cheng M, Hu J, Liu J, Comprehensive identification of immuno-related transcriptional signature for active pulmonary tuberculosis by integrated analysis of array and single cell RNA-seq. J Infect (2022) 85(5):534–44. doi: 10.1016/j.jinf.2022.08.017 36007657

[232] Fox A, Dutt TS, Karger B, Obregón-Henao A, Anderson GB, Henao-Tamayo M. Acquisition of high-quality spectral flow cytometry data. Curr Protoc Cytom (2020) 93(1):e74. doi: 10.1002/cpcy.74 32421215 PMC8801208

[233] Radtke AJ, Chu CJ, Yaniv Z, Yao L, Marr J, Beuschel RT, IBEX: an iterative immunolabeling and chemical bleaching method for high-content imaging of diverse tissues. Nat Protoc (2022) 17(2):378–401. doi: 10.1038/s41596-021-00644-9 35022622 PMC13430508

[234] Gordon KS, Kyung T, Perez CR, Holec PV, Ramos A, Zhang AQ, Screening for CD19-specific chimaeric antigen receptors with enhanced signalling via a barcoded library of intracellular domains. Nat BioMed Eng (2022) 6(7):855–66. doi: 10.1038/s41551-022-00896-0 35710755 PMC9389442

[235] Xu C, He J, Wang H, Zhang Y, Wu J, Zhao L, Single-cell transcriptomic analysis identifies an immune-prone population in erythroid precursors during human ontogenesis. Nat Immunol (2022) 23(7):1109–20. doi: 10.1038/s41590-022-01245-8 35761081

[236] Lin JR, Fallahi-Sichani M, Chen JY, Sorger PK. Cyclic immunofluorescence (CycIF), a highly multiplexed method for single-cell imaging. Curr Protoc Chem Biol (2016) 8(4):251–64. doi: 10.1002/cpch.14 27925668 PMC5233430

[237] Ranzoni AM, Tangherloni A, Berest I, Riva SG, Myers B, Strzelecka PM, Integrative single-cell RNA-seq and ATAC-seq analysis of human developmental hematopoiesis. Cell Stem Cell (2021) 28(3):472–87.e7. doi: 10.1016/j.stem.2020.11.015 33352111 PMC7939551

[238] Ma S, Zhang B, LaFave LM, Earl AS, Chiang Z, Hu Y, Chromatin potential identified by shared single-cell profiling of RNA and chromatin. Cell (2020) 183(4):1103–16.e20. doi: 10.1016/j.cell.2020.09.056 33098772 PMC7669735

[239] Tan WLW, Seow WQ, Zhang A, Rhee S, Wong WH, Greenleaf WJ, Current and future perspectives of single-cell multi-omics technologies in cardiovascular research. Nat Cardiovasc Res (2023) 2(1):20–34. doi: 10.1038/s44161-022-00205-7 39196210 PMC11974510

[240] Eberhardt N, Giannarelli C. How single-cell technologies have provided new insights into atherosclerosis. Arterioscler Thromb Vasc Biol (2022) 42(3):243–52. doi: 10.1161/ATVBAHA.121.315849 35109673 PMC8966900

[241] de Winther MPJ, Bäck M, Evans P, Gomez D, Goncalves I, Jørgensen HF, Translational opportunities of single-cell biology in atherosclerosis. Eur Heart J (2023) 44(14):1216–30. doi: 10.1093/eurheartj/ehac686 36478058 PMC10120164

[242] Skylaki S, Hilsenbeck O, Schroeder T. Challenges in long-term imaging and quantification of single-cell dynamics. Nat Biotechnol (2016) 34(11):1137–44. doi: 10.1038/nbt.3713 27824848

[243] Chang Q, Ornatsky OI, Siddiqui I, Loboda A, Baranov VI, Hedley DW. Imaging mass cytometry. Cytomet A (2017) 91(2):160–9. doi: 10.1002/cyto.a.23053 28160444

[244] Lin JR, Izar B, Wang S, Yapp C, Mei S, Shah PM, Highly multiplexed immunofluorescence imaging of human tissues and tumors using t-CyCIF and conventional optical microscopes. eLife (2018) 7:e31657. doi: 10.7554/eLife.31657 29993362 PMC6075866

[245] Codeluppi S, Borm LE, Zeisel A, La Manno G, van Lunteren JA, Svensson CI, Spatial organization of the somatosensory cortex revealed by osmFISH. Nat Methods (2018) 15(11):932–5. doi: 10.1038/s41592-018-0175-z 30377364

[246] Heath JR, Ribas A, Mischel PS. Single-cell analysis tools for drug discovery and development. Nat Rev Drug Discov (2016) 15(3):204–16. doi: 10.1038/nrd.2015.16 26669673 PMC4883669

[247] Keating SM, Taylor DL, Plant AL, Litwack ED, Kuhn P, Greenspan EJ, Opportunities and challenges in implementation of multiparameter single cell analysis platforms for clinical translation. Clin Transl Sci (2018) 11(3):267–76. doi: 10.1111/cts.12536 29498218 PMC5944591

[248] Gaudillière B, Fragiadakis GK, Bruggner RV, Nicolau M, Finck R, Tingle M, Clinical recovery from surgery correlates with single-cell immune signatures. Sci Transl Med (2014) 6(255):255ra131. doi: 10.1126/scitranslmed.3009701 PMC4334126 25253674

[249] Levitin HM, Yuan J, Sims PA. Single-cell transcriptomic analysis of tumor heterogeneity. Trends Cancer (2018) 4(4):264–8. doi: 10.1016/j.trecan.2018.02.003 29606308 PMC5993208

[250] Rajkomar A, Dean J, Kohane I. Machine learning in medicine. N Engl J Med (2019) 380(14):1347–58. doi: 10.1056/NEJMra1814259 30943338

[251] Nwanosike EM, Conway BR, Merchant HA, Hasan SS. Potential applications and performance of machine learning techniques and algorithms in clinical practice: a systematic review. Int J Med Inform (2022) 159:104679. doi: 10.1016/j.ijmedinf.2021.104679 34990939

[252] Barnett GO, Cimino JJ, Hupp JA, Hoffer EP. DXplain: an evolving diagnostic decision-support system. JAMA (1987) 258(1):67–74. doi: 10.1001/jama.258.1.67 3295316

[253] Kuperman GJ, Gardner RM, Pryor TA. HELP: a dynamic hospital information system. New York, NY: Springer-Verlag (1991).

[254] Johnson KW, Glicksberg BS, Hodos RA, Shameer K, Dudley JT. Causal inference on electronic health records to assess blood pressure treatment targets: an application of the parametric g formula. Pac Symp Biocomput (2018) 23:180–91. doi: 10.1142/9789813235533_0017 29218880 PMC5728675

[255] Shameer K, Johnson KW, Glicksberg BS, Dudley JT, Sengupta PP. Machine learning in cardiovascular medicine: are we there yet? Heart (2018) 104(14):1156–64. doi: 10.1136/heartjnl-2017-311198 29352006

[256] Lopez-Jimenez F, Attia Z, Arruda-Olson AM, Carter R, Chareonthaitawee P, Jouni H, Artificial intelligence in cardiology: present and future. Mayo Clin Proc (2020) 95(5):1015–39. doi: 10.1016/j.mayocp.2020.01.038 32370835

[257] Olsen CR, Mentz RJ, Anstrom KJ, Page D, Patel PA. Clinical applications of machine learning in the diagnosis, classification, and prediction of heart failure. Am Heart J (2020) 229:1–17. doi: 10.1016/j.ahj.2020.07.009 32905873

[258] Rasmy L, Wu Y, Wang N, Geng X, Zheng WJ, Wang F, A study of generalizability of recurrent neural network-based predictive models for heart failure onset risk using a large and heterogeneous EHR data set. J BioMed Inform (2018) 84:11–6. doi: 10.1016/j.jbi.2018.06.011 29908902 PMC6076336

[259] Choi E, Schuetz A, Stewart WF, Sun J. Using recurrent neural network models for early detection of heart failure onset. J Am Med Inform Assoc (2017) 24(2):361–70. doi: 10.1093/jamia/ocw112 27521897 PMC5391725

[260] Raghunath S, Ulloa Cerna AE, Jing L, vanMaanen DP, Stough J, Hartzel DN, Prediction of mortality from 12-lead electrocardiogram voltage data using a deep neural network. Nat Med (2020) 26(6):886–91. doi: 10.1038/s41591-020-0870-z 32393799

[261] Zhao J, Feng Q, Wu P, Lupu RA, Wilke RA, Wells QS, Learning from longitudinal data in electronic health record and genetic data to improve cardiovascular event prediction. Sci Rep (2019) 9(1):717. doi: 10.1038/s41598-018-36745-x 30679510 PMC6345960

[262] Weng SF, Reps J, Kai J, Garibaldi JM, Qureshi N. Can machine-learning improve cardiovascular risk prediction using routine clinical data? PloS One (2017) 12(4):e0174944. doi: 10.1371/journal.pone.0174944 28376093 PMC5380334

[263] Haro Alonso D, Wernick MN, Yang Y, Germano G, Berman DS, Slomka P. Prediction of cardiac death after adenosine myocardial perfusion SPECT based on machine learning. J Nucl Cardiol (2019) 26(5):1746–54. doi: 10.1007/s12350-018-1250-7 29542015 PMC6138585

[264] Hannun AY, Rajpurkar P, Haghpanahi M, Tison GH, Bourn C, Turakhia MP, Cardiologist-level arrhythmia detection and classification in ambulatory electrocardiograms using a deep neural network. Nat Med (2019) 25(1):65–9. doi: 10.1038/s41591-018-0268-3 30617320 PMC6784839

[265] Siontis KC, Noseworthy PA, Attia ZI, Friedman PA. Artificial intelligence-enhanced electrocardiography in cardiovascular disease management. Nat Rev Cardiol (2021) 18(7):465–78. doi: 10.1038/s41569-020-00503-2 33526938 PMC7848866

[266] Arsanjani R, Xu Y, Dey D, Vahistha V, Shalev A, Nakanishi R, Improved accuracy of myocardial perfusion SPECT for detection of coronary artery disease by machine learning in a large population. J Nucl Cardiol (2013) 20(4):553–62. doi: 10.1007/s12350-013-9706-2 23703378 PMC3732038

[267] Siegersma KR, Leiner T, Chew DP, Appelman Y, Hofstra L, Verjans JW. Artificial intelligence in cardiovascular imaging: state of the art and implications for the imaging cardiologist. Neth Heart J (2019) 27(9):403–13. doi: 10.1007/s12471-019-01311-1 31399886 PMC6712136

[268] de Siqueira VS, Borges MM, Furtado RG, Dourado CN, da Costa RM. Artificial intelligence applied to support medical decisions for the automatic analysis of echocardiogram images: a systematic review. Artif Intell Med (2021) 120:102165. doi: 10.1016/j.artmed.2021.102165 34629153

[269] Leclerc S, Smistad E, Pedrosa J, Ostvik A, Cervenansky F, Espinosa F, Deep learning for segmentation using an open large-scale dataset in 2D echocardiography. IEEE Trans Med Imaging (2019) 38(9):2198–210. doi: 10.1109/TMI.2019.2900516 30802851

[270] Madani A, Ong JR, Tibrewal A, Mofrad MRK. Deep echocardiography: data-efficient supervised and semi-supervised deep learning towards automated diagnosis of cardiac disease. NPJ Digit Med (2018) 1:59. doi: 10.1038/s41746-018-0065-x 31304338 PMC6550282

[271] van Velzen SGM, Lessmann N, Velthuis BK, Bank IEM, van den Bongard DHJG, Leiner T, Deep learning for automatic calcium scoring in CT: validation using multiple cardiac CT and chest CT protocols. Radiology (2020) 295(1):66–79. doi: 10.1148/radiol.2020191621 32043947 PMC7106943

[272] Zreik M, van Hamersvelt RW, Khalili N, Wolterink JM, Voskuil M, Viergever MA, Deep learning analysis of coronary arteries in cardiac CT angiography for detection of patients requiring invasive coronary angiography. IEEE Trans Med Imaging (2020) 39(5):1545–57. doi: 10.1109/TMI.2019.2953054 31725371

[273] Zhang N, Yang G, Gao Z, Xu C, Zhang Y, Shi R, Deep learning for diagnosis of chronic myocardial infarction on nonenhanced cardiac cine MRI. Radiology (2019) 291(3):606–17. doi: 10.1148/radiol.2019182304 31038407

[274] Bernard O, Lalande A, Zotti C, Cervenansky F, Yang X, Heng PA, Deep learning techniques for automatic MRI cardiac multi-structures segmentation and diagnosis: is the problem solved? IEEE Trans Med Imaging (2018) 37(11):2514–25. doi: 10.1109/TMI.2018.2837502 29994302

[275] Cikes M, Sanchez-Martinez S, Claggett B, Duchateau N, Piella G, Butakoff C, Machine learning-based phenogrouping in heart failure to identify responders to cardiac resynchronization therapy. Eur J Heart Fail (2019) 21(1):74–85. doi: 10.1002/ejhf.1333 30328654

[276] Segar MW, Patel KV, Ayers C, Basit M, Tang WHW, Willett D, Phenomapping of patients with heart failure with preserved ejection fraction using machine learning-based unsupervised cluster analysis. Eur J Heart Fail (2020) 22(1):148–58. doi: 10.1002/ejhf.1621 31637815

[277] Verdonschot JAJ, Merlo M, Dominguez F, Wang P, Henkens MTHM, Adriaens ME, Phenotypic clustering of dilated cardiomyopathy patients highlights important pathophysiological differences. Eur Heart J (2021) 42(2):162–74. doi: 10.1093/eurheartj/ehaa841 33156912 PMC7813623

[278] Galli E, Bourg C, Kosmala W, Oger E, Donal E. Phenomapping heart failure with preserved ejection fraction using machine learning cluster analysis: prognostic and therapeutic implications. Heart Fail Clin (2021) 17(3):499–518. doi: 10.1016/j.hfc.2021.02.010 34051979

[279] Cheng J, Novati G, Pan J, Bycroft C, Žemgulytė A, Applebaum T, Accurate proteome-wide missense variant effect prediction with AlphaMissense. Science (2023) 381(6664):eadg7492. doi: 10.1126/science.adg7492 37733863

[280] Burris JF, Puglisi JT. Impact of federal regulatory changes on clinical pharmacology and drug development: the common rule and the 21st century cures act. J Clin Pharmacol (2018) 58(3):281–5. doi: 10.1002/jcph.1026 28981164

[281] Spatz ES, Ginsburg GS, Rumsfeld JS, Turakhia MP. Wearable digital health technologies for monitoring in cardiovascular medicine. N Engl J Med (2024) 390(4):346–56. doi: 10.1056/NEJMra2301903 38265646

[282] Ng K, Steinhubl SR, deFilippi C, Dey S, Stewart WF. Early detection of heart failure using electronic health records: practical implications for time before diagnosis, data diversity, data quantity, and data density. Circ Cardiovasc Qual Outcomes (2016) 9(6):649–58. doi: 10.1161/CIRCOUTCOMES.116.002797 28263940 PMC5341145

[283] Deo RC, Nallamothu BK. Learning about machine learning: the promise and pitfalls of big data and the electronic health record. Circ Cardiovasc Qual Outcomes (2016) 9(6):618–20. doi: 10.1161/CIRCOUTCOMES.116.003308 28263936 PMC5832331

[284] Erin Lehr Angelini. IBM Watson health and the broad institute launch initiative to help clinicians predict the risk of cardiovascular disease with genomics and AI. PR Newswire (2019). Available at: https://www.prnewswire.com/news-releases/ibm-watson-health-and-the-broad-institute-launch-initiative-to-help-clinicians-predict-the-risk-of-cardiovascular-disease-with-genomics-and-ai-300794505.html

[285] Agrawal R Microsoft AI network for cardiology with Apollo Hospitals to bring new insights in predicting population-based heart diseases. Microsoft News Center India (2018). Available at: https://news.microsoft.com/en-in/features/microsoft-ai-network-healthcare-apollo-hospitals-cardiac-disease-prediction/

[286] Poplin R, Varadarajan AV, Blumer K, Liu Y, McConnell MV, Corrado GS, Prediction of cardiovascular risk factors from retinal fundus photographs via deep learning. Nat BioMed Eng (2018) 2(3):158–64. doi: 10.1038/s41551-018-0195-0 31015713

[287] Turakhia MP, Desai M, Hedlin H, Rajmane A, Talati N, Ferris T, Rationale and design of a large-scale, app-based study to identify cardiac arrhythmias using a smartwatch: the Apple Heart Study. Am Heart J (2019) 207:66–75. doi: 10.1016/j.ahj.2018.09.002 30392584 PMC8099048

[288] Paul SM, Mytelka DS, Dunwiddie CT, Persinger CC, Munos BH, Lindborg SR, How to improve R&D productivity: the pharmaceutical industry’s grand challenge. Nat Rev Drug Discov (2010) 9(3):203–14. doi: 10.1038/nrd3078 20168317

[289] Sholl DS, Steckel JA. Density functional theory: a practical introduction. Hoboken, NJ: John Wiley & Sons (2009). doi: 10.1002/9780470447710

[290] Yaffe D, Cohen Y, Espinosa G, Arenas A, Giralt F. A fuzzy ARTMAP based on quantitative structure-property relationships (QSPRs) for predicting aqueous solubility of organic compounds. J Chem Inf Comput Sci (2001) 41(5):1177–207. doi: 10.1021/ci010323u 11604019

[291] Verma J, Khedkar VM, Coutinho EC. 3D-QSAR in drug design–a review. Curr Top Med Chem (2010) 10(1):95–115. doi: 10.2174/156802610790232260 19929826

[292] Lamb J, Crawford ED, Peck D, Modell JW, Blat IC, Wrobel MJ, The Connectivity Map: using gene-expression signatures to connect small molecules, genes, and disease. Science (2006) 313(5795):1929–35. doi: 10.1126/science.1132939 17008526

[293] Subramanian A, Narayan R, Corsello SM, Peck DD, Natoli TE, Lu X, A next generation connectivity map: L1000 platform and the first 1,000,000 profiles. Cell (2017) 171(6):1437–52.e17. doi: 10.1016/j.cell.2017.10.049 29195078 PMC5990023

[294] Halu A, Chelvanambi S, Decano JL, Matamalas JT, Whelan M, Asano T, Integrating pharmacogenomics and cheminformatics with diverse disease phenotypes for cell type-guided drug discovery. Genome Med (2025) 17(1):7. doi: 10.1186/s13073-025-01431-x 39833831 PMC11744892

[295] Duan Q, Reid SP, Clark NR, Wang Z, Fernandez NF, Rouillard AD, L1000CDS2: Lincs L1000 characteristic direction signatures search engine. NPJ Syst Biol Appl (2016) 2:16015. doi: 10.1038/npjsba.2016.15 28413689 PMC5389891

[296] Musa A, Ghoraie LS, Zhang SD, Glazko G, Yli-Harja O, Dehmer M, A review of connectivity map and computational approaches in pharmacogenomics. Brief Bioinform (2017) 18(5):903. doi: 10.1093/bib/bbx023 28334173 PMC6113891

[297] Iwata A, Chelvanambi S, Asano T, Whelan M, Nakamura Y, Aikawa E, Gene expression profiles of precursor cells identify compounds that reduce NRP1 surface expression in macrophages: implication for drug repositioning for COVID-19. Front Cardiovasc Med (2024) 11:1438396. doi: 10.3389/fcvm.2024.1438396 39512370 PMC11541348

[298] Swinney DC. Phenotypic vs. target-based drug discovery for first-in-class medicines. Clin Pharmacol Ther (2013) 93(4):299–301. doi: 10.1038/clpt.2012.236 23511784

[299] Swinney DC, Anthony J. How were new medicines discovered? Nat Rev Drug Discov (2011) 10(7):507–19. doi: 10.1038/nrd3480 21701501

[300] Chen R, Liu X, Jin S, Lin J, Liu J. Machine learning for drug-target interaction prediction. Molecules (2018) 23(9):2208. doi: 10.3390/molecules23092208 30200333 PMC6225477

[301] Sachdev K, Gupta MK. A comprehensive review of feature based methods for drug target interaction prediction. J BioMed Inform (2019) 93:103159. doi: 10.1016/j.jbi.2019.103159 30926470

[302] Polishchuk PG, Madzhidov TI, Varnek A. Estimation of the size of drug-like chemical space based on GDB-17 data. J Comput Aid Mol Des (2013) 27(8):675–9. doi: 10.1007/s10822-013-9672-4 23963658

[303] Ezzat A, Wu M, Li XL, Kwoh CK. Computational prediction of drug-target interactions using chemogenomic approaches: an empirical survey. Brief Bioinform (2019) 20(4):1337–57. doi: 10.1093/bib/bby002 29377981

[304] Lim S, Lu Y, Cho CY, Sung I, Kim J, Kim Y, A review on compound-protein interaction prediction methods: data, format, representation and model. Comput Struct Biotechnol J (2021) 19:1541–56. doi: 10.1016/j.csbj.2021.03.004 33841755 PMC8008185

[305] Bagherian M, Sabeti E, Wang K, Sartor MA, Nikolovska-Coleska Z, Najarian K. Machine learning approaches and databases for prediction of drug-target interaction: a survey paper. Brief Bioinform (2021) 22(1):247–69. doi: 10.1093/bib/bbz157 31950972 PMC7820849

[306] Huang K, Fu T, Glass LM, Zitnik M, Xiao C, Sun J. DeepPurpose: a deep learning library for drug-target interaction prediction. Bioinformatics (2021) 36(22–23):5545–7. doi: 10.1093/bioinformatics/btaa1005 33275143 PMC8016467

[307] Cheng Y, Gong Y, Liu Y, Song B, Zou Q. Molecular design in drug discovery: a comprehensive review of deep generative models. Brief Bioinform (2021) 22(6):bbab344. doi: 10.1093/bib/bbab344 34415297

[308] Tong X, Liu X, Tan X, Li X, Jiang J, Xiong Z, Generative models for *de novo* drug design. J Med Chem (2021) 64(19):14011–27. doi: 10.1021/acs.jmedchem.1c00927 34533311

[309] Bian Y, Xie XQ. Generative chemistry: drug discovery with deep learning generative models. J Mol Model (2021) 27(3):71. doi: 10.1007/s00894-021-04674-8 33543405 PMC10984615

[310] Shayakhmetov R, Kuznetsov M, Zhebrak A, Kadurin A, Nikolenko S, Aliper A, Molecular generation for desired transcriptome changes with adversarial autoencoders. Front Pharmacol (2020) 11:269. doi: 10.3389/fphar.2020.00269 32362822 PMC7182000

[311] Méndez-Lucio O, Baillif B, Clevert DA, Rouquié D, Wichard J. *De novo* generation of hit-like molecules from gene expression signatures using artificial intelligence. Nat Commun (2020) 11(1):10. doi: 10.1038/s41467-019-13807-w 31900408 PMC6941972

[312] Jumper J, Evans R, Pritzel A, Green T, Figurnov M, Ronneberger O, Highly accurate protein structure prediction with AlphaFold. Nature (2021) 596(7873):583–9. doi: 10.1038/s41586-021-03819-2 34265844 PMC8371605

[313] Thornton JM, Laskowski RA, Borkakoti N. AlphaFold heralds a data-driven revolution in biology and medicine. Nat Med (2021) 27(10):1666–9. doi: 10.1038/s41591-021-01533-0 34642488

[314] Pirillo A, Catapano AL, Norata GD. Monoclonal antibodies in the management of familial hypercholesterolemia: focus on PCSK9 and ANGPTL3 inhibitors. Curr Atheroscler Rep (2021) 23(12):79. doi: 10.1007/s11883-021-00972-x 34698927 PMC8549899

[315] Békés M, Langley DR, Crews CM. Protac targeted protein degraders: the past is prologue. Nat Rev Drug Discov (2022) 21(3):181–200. doi: 10.1038/s41573-021-00371-6 35042991 PMC8765495

[316] Falese JP, Donlic A, Hargrove AE. Targeting RNA with small molecules: from fundamental principles towards the clinic. Chem Soc Rev (2021) 50(4):2224–43. doi: 10.1039/d0cs01261k 33458725 PMC8018613

[317] Zhu Y, Zhu L, Wang X, Jin H. RNA-based therapeutics: an overview and prospectus. Cell Death Dis (2022) 13(7):644. doi: 10.1038/s41419-022-05075-2 35871216 PMC9308039

[318] Oostveen RF, Khera AV, Kathiresan S, Stroes ESG, Fitzgerald K, Harms MJ, New approaches for targeting PCSK9: small-interfering ribonucleic acid and genome editing. Arterioscler Thromb Vasc Biol (2023) 43(7):1081–92. doi: 10.1161/ATVBAHA.122.317963 37259866

[319] Crooke ST, Baker BF, Crooke RM, Liang XH. Antisense technology: an overview and prospectus. Nat Rev Drug Discov (2021) 20(6):427–53. doi: 10.1038/s41573-021-00162-z 33762737

[320] Pickar-Oliver A, Gersbach CA. The next generation of CRISPR-Cas technologies and applications. Nat Rev Mol Cell Biol (2019) 20(8):490–507. doi: 10.1038/s41580-019-0131-5 31147612 PMC7079207

[321] Zhou J, Rossi J. Aptamers as targeted therapeutics: current potential and challenges. Nat Rev Drug Discov (2017) 16(3):181–202. doi: 10.1038/nrd.2016.199 27807347 PMC5700751

[322] Highleyman L FDA approves fomivirsen, famciclovir, and thalidomide. Food and Drug Administration. Beta (1998) 5 11365993

[323] Yu AM, Tu MJ. Deliver the promise: RNAs as a new class of molecular entities for therapy and vaccination. Pharmacol Ther (2022) 230:107967. doi: 10.1016/j.pharmthera.2021.107967 34403681 PMC9477512

[324] Fogacci F, Ferri N, Toth PP, Ruscica M, Corsini A, Cicero AFG. Efficacy and safety of mipomersen: a systematic review and meta-analysis of randomized clinical trials. Drugs (2019) 79(7):751–66. doi: 10.1007/s40265-019-01114-z 30989634

[325] Ray KK, Wright RS, Kallend D, Koenig W, Leiter LA, Raal FJ, Two phase 3 trials of inclisiran in patients with elevated LDL cholesterol. N Engl J Med (2020) 382(16):1507–19. doi: 10.1056/NEJMoa1912387 32187462

[326] Tsimikas S, Karwatowska-Prokopczuk E, Gouni-Berthold I, Tardif JC, Baum SJ, Steinhagen-Thiessen E, Lipoprotein(a) reduction in persons with cardiovascular disease. N Engl J Med (2020) 382(3):244–55. doi: 10.1056/NEJMoa1905239 31893580

[327] Kristen AV, Ajroud-Driss S, Conceição I, Gorevic P, Kyriakides T, Obici L. Patisiran, an RNAi therapeutic for the treatment of hereditary transthyretin-mediated amyloidosis. Neurodegener Dis Manag (2019) 9(1):5–23. doi: 10.2217/nmt-2018-0033 30480471

[328] Solomon SD, Adams D, Kristen A, Grogan M, González-Duarte A, Maurer MS, Effects of patisiran, an RNA interference therapeutic, on cardiac parameters in patients with hereditary transthyretin-mediated amyloidosis. Circulation (2019) 139(4):431–43. doi: 10.1161/CIRCULATIONAHA.118.035831 30586695 PMC12611557

[329] Seal RL, Chen LL, Griffiths-Jones S, Lowe TM, Mathews MB, O’Reilly D, A guide to naming human non-coding RNA genes. EMBO J (2020) 39(6):e103777. doi: 10.15252/embj.2019103777 32090359 PMC7073466

[330] Uchida S, Adams JC. Physiological roles of non-coding RNAs. Am J Physiol Cell Physiol (2019) 317(1):C1–2. doi: 10.1152/ajpcell.00114.2019 31091141 PMC6689750

[331] Das S, Shah R, Dimmeler S, Freedman JE, Holley C, Lee JM, Noncoding RNAs in cardiovascular disease: current knowledge, tools and technologies for investigation, and future directions: a scientific statement from the American Heart Association. Circ Genom Precis Med (2020) 13(4):e000062. doi: 10.1161/HCG.0000000000000062 32812806

[332] Laggerbauer B, Engelhardt S. MicroRNAs as therapeutic targets in cardiovascular disease. J Clin Invest (2022) 132(11):e159179. doi: 10.1172/JCI159179 35642640 PMC9151703

[333] Winkle M, El-Daly SM, Fabbri M, Calin GA. Noncoding RNA therapeutics - challenges and potential solutions. Nat Rev Drug Discov (2021) 20(8):629–51. doi: 10.1038/s41573-021-00219-z 34145432 PMC8212082

[334] Chen Y, Li Z, Chen X, Zhang S. Long non-coding RNAs: from disease code to drug role. Acta Pharm Sin B (2021) 11(2):340–54. doi: 10.1016/j.apsb.2020.10.001 33643816 PMC7893121

[335] Uchida S, Dimmeler S. Long noncoding RNAs in cardiovascular diseases. Circ Res (2015) 116(4):737–50. doi: 10.1161/CIRCRESAHA.116.302521 25677520

[336] Yeh CF, Chang YE, Lu CY, Hsuan CF, Chang WT, Yang KC. Expedition to the missing link: long noncoding RNAs in cardiovascular diseases. J BioMed Sci (2020) 27(1):48. doi: 10.1186/s12929-020-00647-w 32241300 PMC7114803

[337] Ezike TC, Okpala US, Onoja UL, Nwike CP, Ezeako EC, Okpara OJ, Advances in drug delivery systems, challenges and future directions. Heliyon (2023) 9(6):e17488. doi: 10.1016/j.heliyon.2023.e17488 37416680 PMC10320272

[338] Vargason AM, Anselmo AC, Mitragotri S. The evolution of commercial drug delivery technologies. Nat BioMed Eng (2021) 5(9):951–67. doi: 10.1038/s41551-021-00698-w 33795852

[339] Paunovska K, Loughrey D, Dahlman JE. Drug delivery systems for RNA therapeutics. Nat Rev Genet (2022) 23(5):265–80. doi: 10.1038/s41576-021-00439-4 34983972 PMC8724758

[340] Spicer AJ, Colcomb PA, Kraft A. Mind the gap: closing the growing chasm between academia and industry. Nat Biotechnol (2022) 40(11):1693–6. doi: 10.1038/s41587-022-01543-4 36347973

[341] Frye S, Crosby M, Edwards T, Juliano R. US academic drug discovery. Nat Rev Drug Discov (2011) 10(6):409–10. doi: 10.1038/nrd3462 21629285 PMC4461005

[342] Huryn DM, Resnick LO, Wipf P. Contributions of academic laboratories to the discovery and development of chemical biology tools. J Med Chem (2013) 56(18):7161–76. doi: 10.1021/jm400132d 23672690 PMC3785552

[343] Brigham and Women’s Hospital. New lab brings BWH, Japanese researchers together [online] (2010). Available at: https://www.bwhpublicationsarchives.org/DisplayCRN.aspx?articleid=1837

[344] Brigham and Women’s Hospital. A $75 million ‘brave idea’ to end coronary heart disease [online] (2010). Available at: https://give.brighamandwomens.org/brave-idea-end-coronary-heart-disease/

[345] Munos BH, Chin WW. How to revive breakthrough innovation in the pharmaceutical industry. Sci Transl Med (2011) 3(89):89cm16. doi: 10.1126/scitranslmed.3002273 21715677

[346] Yildirim O, Gottwald M, Schüler P, Michel MC. Opportunities and challenges for drug development: public-private partnerships, adaptive designs and big data. Front Pharmacol (2016) 7:461. doi: 10.3389/fphar.2016.00461 27999543 PMC5138214

[347] Nwaka S, Ridley RG. Virtual drug discovery and development for neglected diseases through public-private partnerships. Nat Rev Drug Discov (2003) 2(11):919–28. doi: 10.1038/nrd1230 14668812

[348] Tralau-Stewart CJ, Wyatt CA, Kleyn DE, Ayad A. Drug discovery: new models for industry-academic partnerships. Drug Discov Today (2009) 14(1–2):95–101. doi: 10.1016/j.drudis.2008.10.003 18992364

[349] Ferrero E, Brachat S, Jenkins JL, Marc P, Skewes-Cox P, Altshuler RC, Ten simple rules to power drug discovery with data science. PloS Comput Biol (2020) 16(8):e1008126. doi: 10.1371/journal.pcbi.1008126 32853229 PMC7451597

[350] Jodogne S Client-side application of deep learning models through teleradiology. Stud Health Technol Inform (2023) 302:997–1001. doi: 10.3233/SHTI230325 37203552

[351] Dong E, Du H, Gardner L. An interactive web-based dashboard to track COVID-19 in real time. Lancet Infect Dis (2020) 20(5):533–4. doi: 10.1016/S1473-3099(20)30120-1 32087114 PMC7159018

[352] Cauchemez S, Cossu G, Delzenne N, Elinav E, Fassin D, Fischer A, Standing the test of COVID-19: charting the new frontiers of medicine. Front Sci (2024) 2:1236919. doi: 10.3389/fsci.2024.1236919

[353] Moreira ED Jr, Kitchin N, Xu X, Dychter SS, Lockhart S, Gurtman A, Safety and efficacy of a third dose of BNT162b2 Covid-19 vaccine. N Engl J Med (2022) 386(20):1910–21. doi: 10.1056/NEJMoa2200674 35320659 PMC9006787

[354] Arbel R, Hammerman A, Sergienko R, Friger M, Peretz A, Netzer D, BNT162b2 vaccine booster and mortality due to Covid-19. N Engl J Med (2021) 385(26):2413–20. doi: 10.1056/NEJMoa2115624 34879190 PMC8728797

[355] Weinreich DM, Sivapalasingam S, Norton T, Ali S, Gao H, Bhore R, REGEN-COV antibody combination and outcomes in outpatients with Covid-19. N Engl J Med (2021) 385(23):e81. doi: 10.1056/NEJMoa2108163 34587383 PMC8522800

[356] O’Brien MP, Forleo-Neto E, Sarkar N, Isa F, Hou P, Chan KC, Effect of subcutaneous casirivimab and imdevimab antibody combination vs placebo on development of symptomatic COVID-19 in early asymptomatic SARS-CoV-2 infection: a randomized clinical trial. JAMA (2022) 327(5):432–41. doi: 10.1001/jama.2021.24939 35029629 PMC8808333

[357] Alameh MG, Tombácz I, Bettini E, Lederer K, Sittplangkoon C, Wilmore JR, Lipid nanoparticles enhance the efficacy of mRNA and protein subunit vaccines by inducing robust T follicular helper cell and humoral responses. Immunity (2022) 55(6):1136–8. doi: 10.1016/j.immuni.2022.05.007 35704995 PMC9195404

[358] Karikó K, Whitehead K, van der Meel R. What does the success of mRNA vaccines tell us about the future of biological therapeutics? Cell Syst (2021) 12(8):757–8. doi: 10.1016/j.cels.2021.07.005 34411542 PMC8372463

[359] Ward B, Yombi JC, Balligand JL, Cani PD, Collet JF, de Greef J, HYGIEIA: hypothesizing the genesis of infectious diseases and epidemics through an integrated systems biology approach. Viruses (2022) 14(7):1373. doi: 10.3390/v14071373 35891354 PMC9318602

[360] Sun J, Aikawa M, Ashktorab H, Beckmann ND, Enger ML, Espinosa JM, A multi-omics strategy to understand PASC through the RECOVER cohorts: a paradigm for a systems biology approach to the study of chronic conditions. Front Syst Biol (2025) 4:1422384. doi: 10.3389/fsysb.2024.1422384 40809128 PMC12342036

[361] Reese JT, Blau H, Casiraghi E, Bergquist T, Loomba JJ, Callahan TJ, Generalisable long COVID subtypes: findings from the NIH N3C and RECOVER programmes. EBiomedicine (2023) 87:104413. doi: 10.1016/j.ebiom.2022.104413 36563487 PMC9769411

[362] Alfano V, Ercolano S. The efficacy of lockdown against COVID-19: a cross-country panel analysis. Appl Health Econ Health Policy (2020) 18(4):509–17. doi: 10.1007/s40258-020-00596-3 32495067 PMC7268966

[363] García-Basteiro AL, Legido-Quigley H. Evaluation of the COVID-19 response in Spain: principles and requirements. Lancet Public Health (2020) 5(11):e575. doi: 10.1016/S2468-2667(20)30208-5 32971009 PMC7505572

[364] Thawonmas R, Hashizume M, Kim Y. Projections of temperature-related suicide under climate change scenarios in Japan. Environ Health Perspect (2023) 131(11):117012. doi: 10.1289/EHP11246 37995154 PMC10666824

[365] Agache I, Akdis C, Akdis M, Al-Hemoud A, Annesi-Maesano I, Balmes J, Immune-mediated disease caused by climate change-associated environmental hazards: mitigation and adaptation. Front Sci (2024) 2:1279192. doi: 10.3389/fsci.2024.1279192 40444110 PMC12121949

[366] Anonymous. The best medicine for improving global health? Reduce inequality. Nature (2023) 619:221. doi: 10.1038/d41586-023-02251-y 37433936

[367] Fuster V, Vedanthan R. Cardiovascular disease and the UN Millennium Development Goals: time to move forward. Nat Clin Pract Cardiovasc Med (2008) 5(10):593. doi: 10.1038/ncpcardio1353 18813335

[368] MalariaWorld. The best medicine for improving global health? Reduce inequality [online] (2023). Available at: https://malariaworld.org/blogs/the-best-medicine-for-improving-global-health-reduce-inequality 10.1038/d41586-023-02251-y 37433936

[369] World Health Organization. Global action plan for the prevention and control of noncommunicable diseases 2013–2020. Geneva: WHO (2013). Available at: https://www.who.int/publications/i/item/9789241506236

[370] World Health Organization. Invisible numbers: the true extent of noncommunicable diseases and what to do about them. Geneva: WHO (2022). Available at: https://www.who.int/publications/i/item/9789240057661

[371] Kones R, Rumana U. Cardiometabolic diseases of civilization: history and maturation of an evolving global threat. An update and call to action. Ann Med (2017) 49(3):260–74. doi: 10.1080/07853890.2016.1271957 27936950

[372] Mocumbi AO. Cardiovascular health care in low- and middle-income countries. Circulation (2024) 149(8):557–9. doi: 10.1161/CIRCULATIONAHA.123.065717 38377254

[373] Qureshi NQ, Mufarrih SH, Bloomfield GS, Tariq W, Almas A, Mokdad AH, Disparities in cardiovascular research output and disease outcomes among high-, middle- and low-income countries - an analysis of global cardiovascular publications over the last decade (2008–2017). Glob Heart (2021) 16(1):4. doi: 10.5334/gh.815 33598384 PMC7845477

[374] Adeyi O, Yadav P, Kazatchkine M. Frontiers of medicine unveiled: equitable access is an imperative. Front Sci (2024) 2:1422583. doi: 10.3389/fsci.2024.1422583

[375] Global Preparedness Monitoring Board. A fragile state of preparedness: 2023 report on the state of the world’s preparedness. Geneva: World Health Organization (2023). Available at: https://www.gpmb.org/reports/m/item/a-fragile-state-of-preparedness-2023-report-on-the-state-of-the-worlds-preparedness

[376] World Health Organization. Pandemic prevention, preparedness and response agreement [online] (2025). Available at: https://www.who.int/news-room/questions-and-answers/item/pandemic-prevention-preparedness-and-response-accord

[377] Dzau V, Fuster V, Frazer J, Snair M. Investing in global health for our future. N Engl J Med (2017) 377(13):1292–6. doi: 10.1056/NEJMsr1707974 28953444

[378] Davis A, Asma S, Blecher M, Benn C, Ezoe S, Fogstad H, Investing in the future of global health. Lancet (2024) 404(10462):1500–3. doi: 10.1016/S0140-6736(24)02191-3 39419053

[379] Estapé Senti M, Ceccaldi A, Luciani M, Saber N, Schurmann PJL, Geerlings MW, NANOSPRESSO: toward personalized, locally produced nucleic acid nanomedicines. Front Sci (2025) 3:1458636. doi: 10.3389/fsci.2025.1458636

